# Genetics in the Ocean's Twilight Zone: Population Structure of the Glacier Lanternfish Across Its Distribution Range

**DOI:** 10.1111/eva.70032

**Published:** 2024-11-06

**Authors:** María Quintela, Eva García‐Seoane, Geir Dahle, Thor A. Klevjer, Webjørn Melle, Roger Lille‐Langøy, François Besnier, Konstantinos Tsagarakis, Maxime Geoffroy, Naiara Rodríguez‐Ezpeleta, Eugenie Jacobsen, David Côté, Sofie Knutar, Laila Unneland, Espen Strand, Kevin Glover

**Affiliations:** ^1^ Population Genetics Group Institute of Marine Research Bergen Norway; ^2^ Plankton Group Institute of Marine Research Bergen Norway; ^3^ Sustainable Oceans and Coasts Møreforsking AS Ålesund Norway; ^4^ Hellenic Centre for Marine Research Institute of Marine Biological Resources and Inland Waters Athens Greece; ^5^ Centre for Fisheries Ecosystems Research Fisheries and Marine Institute of Memorial University of Newfoundland and Labrador St. John's Newfoundland and Labrador Canada; ^6^ Faculty of Biosciences, Fisheries and Economics UiT the Arctic University of Norway Tromsø Norway; ^7^ AZTI Basque Research and Technology Alliance (BRTA) Marine Research Sukarrieta Spain; ^8^ Northwest Atlantic Fisheries Centre Fisheries and Oceans Canada St. John's Canada

**Keywords:** Atlantic, *Benthosema glaciale*, chromosome inversion, genetic structure, glacier lanternfish, Mediterranean, mesopelagic fish, SNPs

## Abstract

The mesopelagic zone represents one of the few habitats that remains relatively untouched from anthropogenic activities. Among the many species inhabiting the north Atlantic mesopelagic zone, glacier lanternfish (*Benthosema glaciale*) is the most abundant and widely distributed*.* This species has been regarded as a potential target for a dedicated fishery despite the scarce knowledge of its population genetic structure. Here, we investigated its genetic structure across the North Atlantic and into the Mediterranean Sea using 121 SNPs, which revealed strong differentiation among three main groups: the Mediterranean Sea, oceanic samples, and Norwegian fjords. The Mediterranean samples displayed less than half the genetic variation of the remaining ones. Very weak or nearly absent genetic structure was detected among geographically distinct oceanic samples across the North Atlantic, which contrasts with the low motility of the species. In contrast, a longitudinal gradient of differentiation was observed in the Mediterranean Sea, where genetic connectivity is known to be strongly shaped by oceanographic processes such as current patterns and oceanographic discontinuities. In addition, 12 of the SNPs, in linkage disequilibrium, drove a three clusters' pattern detectable through Principal Component Analysis biplot matching the genetic signatures generally associated with large chromosomal rearrangements, such as inversions. The arrangement of this putative inversion showed frequency differences between open‐ocean and more confined water bodies such as the fjords and the Mediterranean, as it was fixed in the latter for the second most common arrangement of the fjord's samples. However, whether genetic differentiation was driven by local adaptation, secondary contact, or a combination of both factors remains undetermined. The major finding of this study is that *B. glaciale* in the North Atlantic‐Mediterranean is divided into three major genetic units, information that should be combined with demographic properties to outline the management of this species prior to any eventual fishery attempt.

## 1 Introduction

Large, sustainable sources of nutritious food are in increasing demand for the expanding world population. The high content of proteins and omega‐3 lipids Eicosapentaenoic acid (EPA) and Docosahexaenoic acid (DHA) present in fish and other marine organisms has led to over a third of the worldwide fishing resources being harvested beyond their biologically sustainable levels (FAO 2020), which fuelled the search for alternatives such as fish in the mesopelagic zone.

The mesopelagic zone, also known as the ocean's twilight zone, is situated between 200–1000 m deep, and, despite hosting up to 90% of the total oceanic fish biomass (Irigoien et al. 2014), knowledge about mesopelagic organisms relative to their trophic interactions, life histories, behaviour, biomass, or population genetic structure is still limited (Hidalgo and Browman 2019; Martin et al. 2020; Standal and Grimaldo 2020). Mesopelagic fish inhabiting this largely unexplored area, which accounts for 20% of the total world's ocean volume, are believed to be the largest unexploited living resource on Earth. Large uncertainties exist regarding stock abundances, with the most ambitious estimates suggesting that the mesopelagic zone could contain around 10 Gt (10,000 million tons) of fish biomass (Fjeld et al. 2023). In spite of mesopelagic fish being deemed possible targets for industrial fisheries since the 1970s due to their widespread distribution and high local concentrations, only sporadic attempts have been made to exploit these resources thus far (figure 3 in Caiger, Lefebve, and Llopiz (2021)), and therefore their stocks remain relatively intact for now.

The family Myctophidae, commonly called lanternfish, is one of the most abundant mesopelagic fish and represents the largest biomass of any vertebrate (Van Noord 2013). Myctophids are a geographically widespread (Catul, Gauns, and Karuppasamy 2011; Gaither et al. 2016) and extremely diverse taxon that comprises some 250 species distributed within 33 genera (Eschmeyer, Fricke, and Van Der Laan 2018). Myctophids conduct diel vertical migrations (DVM) (Olivar et al. 2012) rising from the twilight zone at night to follow and feed on their zooplankton prey, therefore actively exporting carbon from the surface to deeper water masses and contributing to the biological pump (Hidaka et al. 2001; Shreeve et al. 2009; Robinson et al. 2010). Myctophids also play a crucial role in energy transfer within pelagic ecosystems by linking planktonic organisms, such as copepods, ostracods, and larvaceans, to top predators (Cherel et al. 2010), including fish (Walker and Nichols 1993), squids (Parry 2006), seabirds (Hedd et al. 2009), and marine mammals (Ohizumi et al. 2003).

The glacier lanternfish *Benthosema glaciale* (Reinhardt 1837), which can grow up to ca. 10 cm (Hulley 1984), has a primarily North amphi‐Atlantic distribution. In the East Atlantic, it ranges from the Barents Sea down to Guinea and into the Mediterranean, whereas on the West it is distributed from Baffin Bay and Greenland to the edge of the Gulf stream. As such, it is the most abundant myctophid in the Atlantic Ocean north of 35°N (Gjøsæter 1973; Chawarski et al. 2022; Olivar et al. 2022; Knutsen et al. 2023). Glacier lanternfish also occurs in Norwegian fjords (Gjøsæter 1973; Gjøsæter and Kawaguchi 1980; Hulley 1984) and has recently been discovered on the continental slope of the Pacific Arctic (Zhang et al. 2022). It is preyed on by different large pelagic fish such as Atlantic mackerel (Walker and Nichols 1993), tuna (Pusineri et al. 2005), and swordfish (Chancollon, Pusineri, and Ridoux 2006), which explains why it stays in the dark mesopelagic zone during the daytime to avoid visual predators.

Spatial variations in meristic characters, growth, and life history parameters have been described for *B. glaciale* (Gjøsæter 1973; Badcock 1981; Kawaguchi and Mauchline 1982). Gjøsæter (1981) reported differentiation between oceanic and fjord samples based on otolith characteristics, length‐weight relationship, and growth parameters. Badcock (1981) reported marked differences in meristic characters between the Mediterranean Sea and the Atlantic populations. Although the mechanisms responsible for such differences have not been elucidated, it has been suggested that the hydrography of partially enclosed seas may provide sufficient barriers to gene flow such that populations within seas may be diverging from those outside the seas (Gartner 1993). The size at sexual maturity and maximum size of mesopelagic fishes in partially enclosed seas is smaller than of their open‐water Atlantic counterparts (Gartner 1991). To our knowledge the hypothesis of different populations of glacier lanternfish across different habitats has never been tested with molecular genetic methods; however, there is vast evidence of genetically‐identified ecotypic differentiation in other marine taxa either driven by bathymetry as in beaked redfish (*Sebastes mentella*) (Benestan et al. 2021), salinity as in European sprat (*Sprattus sprattus*) (Quintela et al. 2020; Pettersson et al. 2024), and marine versus coastal habitat such as in European anchovy (*Engraulis encrasicolus*) (Le Moan, Gagnaire, and Bonhomme 2016), long‐snouted seahorse (*Hyppocampus guttulatus*) (Riquet et al. 2019; Meyer et al. 2024), northern shrimp (*Pandalus borealis*) (Hansen et al. 2021) or Atlantic cod (*Gadus morhua*) (Knutsen et al. 2018), to mention a few.

Establishing sustainable fisheries to commercially exploit marine resources, including *B. glaciale*, requires a number of conditions to be fulfilled. One pre‐requisite is that population genetic structure is understood to ensure that the resource is appropriately split into biologically correct management units or stocks to prevent the overexploitation of unique spawning components (e.g., see Reiss et al. 2009; Kerr et al. 2017 for revision). These stocks can be influenced by species range shifts associated with climate change (Palacios‐Abrantes et al. 2022; Dahms and Killen 2023). The separation of populations may also be important when interpreting and analysing key demographic parameters such as growth rate, fecundity, size at first reproduction, and maximum size, which may be influenced by genetic composition and local environmental conditions alike. The genetic component should therefore be incorporated when attempting to outline management areas, as locally‐adapted populations or subdivided ones might both have different sustainable yield levels and be more prone to the negative effects of overfishing (Waples, Punt, and Cope 2008; Pinsky and Palumbi 2014). In addition, historical barriers, such as past isolated stocks that are now in secondary contact, can also inflate the population structure and often mimic the patterns of differentiation expected from physical and/or environmental barriers, as it has been described in ecotypes of European anchovy (Le Moan, Gagnaire, and Bonhomme 2016). Here we present the first study of the population genetic structure of the glacier lanternfish across the North Atlantic, including the Norwegian fjords and the Mediterranean Sea. We further test the hypothesis that physical barriers between the open‐ocean and confined water bodies result in different population structures in mesopelagic fish.

## 2 Materials and Methods

### 2.1 Sampling and Genotyping

**FIGURE 1:**
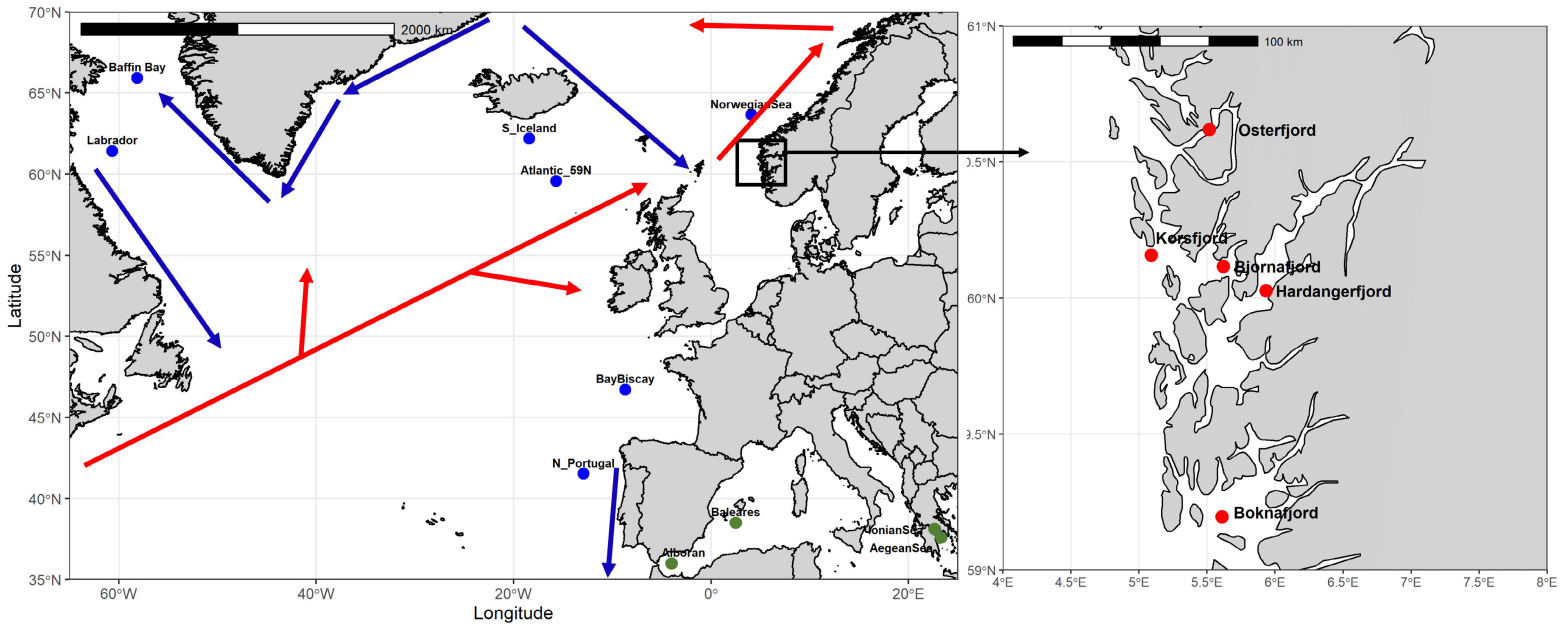
*Benthosema glaciale* sampling regions. Blue dots depict the Ocean samples, whereas red and green dots depict the fjords and the Mediterranean, respectively. The main cold ocean currents are outlined in blue, whereas the warm ones are outlined in red. As some of the sampling sites consist of different nearby stations, the geographic coordinates indicated per sample are an average of all trawls for simplicity.

In the period 2017–2022, a total of 1419 individuals was collected in 16 locations, covering a large proportion of the distribution range of the species. Sampling included the NW (Labrador and Baffin Bay) and the NE (South Iceland to the North of Portugal) Atlantic regions, including five fjords on the SW coast of Norway, as well as the Western (Alborán Sea) and Eastern (Ionian and Aegean Seas) Mediterranean Seas (Figure 1). The Mediterranean samples from the Ionian Sea come from the very enclosed, deep, and isolated gulf of Corinth, which has geophysical features similar to the Norwegian fjords (Kapelonis et al. 2023). Sampling depth varied both in time of the day and location, from 60 m at night inside the Norwegian fjords, down to 570 m in the Ionian Sea, and up to 1100 m in the Atlantic. Fin clips were taken and stored in ethanol 96° prior to DNA isolation, which was conducted using SPRI paramagnetic beads from the Beckman Coulter DNAdvance kit (A48706). DNA concentration was quantified using NanoDrop 8000.

Sampling sites were located in three putatively different habitats based upon former knowledge on other marine fish (e.g., Quintela et al. 2020)*:* Norwegian fjords, Mediterranean Sea, and open Atlantic Ocean. Four out of the 16 sites were selected for SNP mining: Korsfjorden (fjords), Alborán (Mediterranean), and two locations in the open ocean at 41 °N and 59 °N, respectively, to account for geographic distance. DNA concentration in these locations was quantified using Thermo Fisher Qubit dsDNA Broad Ranger (Q32853), and DNA from 10 individuals per site was pooled, and one library was done per pool using Illumina TrueSeq DNA Nano. Pooled samples were sequenced on a NovaSeq 6000 using SP flow cell configuration (150 PE). FASTQC v0.11.5 (https://www.bioinformatics.babraham.ac.uk/projects/fastqc/) and MULTIQC v1.7 (https://github.com/MultiQC/MultiQC) were used for data preprocessing, which included deduplication, adaptor removal, and filtering overrepresented sequences. Pool‐sequencing reads were aligned to the existing draft assembly for *B. glaciale* obtained from the NCBI repository (https://www.ncbi.nlm.nih.gov/assembly/GCA_900323375.1/), consisting of 676,314,385 base pairs distributed on 188,319 contigs. Read alignment was performed with BWA V0.7.17 (Li and Durbin 2010) using default parameters, and variant sites were called using the mpileup function (Li 2011) from Samtools V1.9 (Li et al. 2009). Variant sites were then filtered for the so‐called quality score (QUAL > 600), minimum and maximum coverage of respectively 10× and 50× in each sample, and SNPs located less than 200 bases from another polymorphic site (SNP or indel) were removed to keep only SNPs with stable primer sequences. Finally, to reduce the probability of selecting linked markers, we retained only one SNP per contig. Likewise, no attempt at selecting diagnostic SNPs to discriminate putative habitats was conducted. A total of circa 2000 SNPs fulfilled those criteria, but the interest was to genotype as many individuals as possible across the species' distribution range using a high‐throughput SNP genotyping approach in an affordable manner. Therefore, a suite of 148 SNPs was randomly selected from the 2000 available; primers were designed, arranged into six multiplex reactions, and genotyped on the 1419 individuals distributed into 16 samples using the Sequenom MassARRAY iPLEX Platform as described by Gabriel, Ziaugra, and Tabbaa (2009).

### 2.2 Genetic Structure

Statistical analyses were restricted to a subset of 121 well‐functioning polymorphic loci obtained out of the 148 screened ones (17 loci were dismissed due to ≥ 25% missing data and 10 were dismissed due to showing allele frequency of ≥ 95% in all samples). Even though the threshold of acceptance of missing data per individual was 25%, only 2.7% of the 1288 finally retained individuals showed missing data > 20%, whereas 63% of them displayed ≤ 5%. To assess if the 121 SNPs would accurately discriminate between individuals in a population, the genotype accumulation curve was built using the function *genotype_curve* in the R (R Core Team 2020) package *poppr* (Kamvar, Tabima, and Grünwald 2014) by randomly sampling × loci without replacement and counting the number of observed multilocus genotypes (MLGs). This repeated *r* times for 1 locus up to *n*‐1 loci, creating *n*‐1 distributions of observed MLGs. The observed (*H*_o_) and unbiased expected heterozygosity (*uH*_e_) as well as the inbreeding coefficient (*F*_IS_) were computed for each sample with GenAlEx v6.1 (Peakall and Smouse 2006). Likewise, the genotype frequency of each locus and its direction (heterozygote deficit or excess) was compared with Hardy–Weinberg expectations (HWE) using the program GENEPOP 4.0.6 (Rousset 2008), as was linkage disequilibrium (LD) between pairwise loci. To test the null hypothesis of unlinked loci expected in sexually recombining populations, the index of association (IA) (Brown, Feldman, and Nevo 1980) and the alternative index, rd (less biased to the number of loci) (Agapow and Burt 2001), were calculated with the R package *poppr* (Kamvar, Tabima, and Grünwald 2014) to assess the multilocus genotypic LD, with disequilibrium indicated when IA and rd differ significantly from 0. The False Discovery Rate (FDR) correction of Benjamini and Hochberg (1995) was applied to *p*‐values to control for Type I errors. Non‐parametric Kruskal–Wallis rank sum test was applied to perform comparisons of estimates of genetic diversity among groups.

Genetic structure was assessed using the Analysis of Molecular Variance (AMOVA) and pairwise *F*_ST_ (Weir and Cockerham 1984), both computed with Arlequin v.3.5.1.2 (Excoffier, Laval, and Schneider 2005). Furthermore, the relationship among samples was examined using the Discriminant Analysis of Principal Components (DAPC) (Jombart, Devillard, and Balloux 2010) implemented in the R (R Core Team 2020) package *agreement* (Jombart 2008), in which groups were defined using geographically explicit locations. To avoid overfitting, both the optimal number of principal components and discriminant functions to be retained were determined using the cross‐validation function (Jombart and Collins 2015; Miller, Cullingham, and Peery 2020). Likewise, a Principal Component Analysis (PCA) with no a priori grouping of samples per location was conducted using the function *dudi.pca* in *ade4* (Dray and Dufour 2007) after replacing missing data with the mean allele frequencies, using no scaled allele frequencies (scale = FALSE). In addition, the Bayesian clustering approach implemented in STRUCTURE v.2.3.4 (Pritchard, Stephens, and Donnelly 2000) and conducted using the software ParallelStructure (Besnier and Glover, 2013) was used to identify genetic groups under a model assuming admixture and correlated allele frequencies without using LOCPRIORS. Ten runs with a burn‐in period consisting of 100,000 replications and a run length of 1000,000 MCMC iterations were performed for *K* = 1 to *K* = 10 clusters. To determine the number of genetic groups, structure output was analysed using two approaches: (a) the *ad hoc* summary statistic Δ*K* of Evanno et al. (2005), and (b) the Puechmaille (2016) four statistics (MedMedK, MedMeanK, MaxMedK, and MaxMeanK), both implemented in StructureSelector (Li and Liu 2018). Finally, the 10 runs for the selected *K*s were averaged with CLUMPP v.1.1.1 (Jakobsson and Rosenberg 2007) using the FullSearch algorithm and the G' pairwise matrix similarity statistic and graphically displayed using bar plots.

### 2.3 Haplotype Groups and LD‐Pruned Analyses

Fisher's exact tests of linkage disequilibrium were calculated between each pair of SNP loci, and the resulting *p*‐values were visualized in the form of a pairwise matrix. This analysis served a double purpose: first to assess if loci driving the PCA pattern (and identifiable through the loadings on the first axis) were linked and, secondly, to LD‐prune the dataset and repeat the analyses aiming to describe genetic structure and outlier detection. The most relevant loci accounting for the striation in the PCA were used to reconstruct the corresponding haplotypes using PHASE v 2.1 (Stephens, Smith, and Donnelly 2004).

## 3 Results

### 3.1 Genetic Structure

Poolseq data revealed significant differentiation among the three putative habitats, differentiation that was particularly strong in the comparisons between oceanic locations and the Mediterranean. However, no differentiation whatsoever was detected between the two oceanic samples (Figure S1).

The resolution power of the SNP array used was evidenced by the plateau of the genotype accumulation curve reached with less than one quarter of the 121 polymorphic loci, meaning that 25–30 SNPs were enough to differentiate unique individuals (Figure S2). Genetic diversity, as determined by the percentage of polymorphic loci or observed and expected heterozygosity, was more than double in the Atlantic Ocean and Norwegian fjords samples compared to all samples from the Mediterranean (Table 1), Kruskal–Wallis *p* < 0.002 for the three estimates. Likewise, samples from the Mediterranean showed slightly fewer loci deviating from HWE, but more distinctly, LD was ~30‐fold lower than in the Ocean or fjord samples.

**TABLE 1:** Sample summary statistics obtained for the set of 121 SNP loci: Sampling sites with geographic coordinates in decimal degrees; sampling depth, number of individuals (*N*), proportion of polymorphic loci, observed heterozygosity, *H*_o_ (mean ± SE); unbiased expected heterozygosity, u*H*_e_ (mean ± SE); inbreeding coefficient, *F*_IS_ (mean ± SE); number of deviations from Hardy–Weinberg equilibrium (HWE) and number of deviations from Linkage Disequilibrium (LD) at *α* = 0.05 both before and (after) False Discovery Rate (FDR) correction. As some of the sampling sites consist of different nearby stations, the geographic coordinates indicated per sample are an average of all trawls for simplicity.

Type	Sample	Year	Latitude	Longitude	Depth (m)	*N*	Polym loci (%)	*H*_o_	*H*_e_	*F*_IS_	Dev HWE (FDR)	Dev LD (FDR)
Ocean	Baffin Bay	2021	65.93	−58.12		43	93.4	0.263 ± 0.016	0.276 ± 0.015	0.031 ± 0.022	16 (9)	517 (485)
	Labrador	2022	61.43	−60.65		37	89.3	0.258 ± 0.018	0.262 ± 0.015	0.028 ± 0.024	13 (4)	458 (429)
	Norwegian Sea	2020	63.65	4.10	650	36	87.6	0.284 ± 0.018	0.288 ± 0.015	0.026 ± 0.026	14 (7)	508 (469)
	S. Iceland	2018–2021	62.19	−18.42		87	90.1	0.294 ± 0.017	0.291 ± 0.015	−0.010 ± 0.018	17 (7)	786 (744)
	Atlantic 59 N	2018–2021	59.58	−15.68	1050–1108	189	95.0	0.289 ± 0.016	0.293 ± 0.015	0.015 ± 0.016	28 (17)	947 (906)
	Bay of Biscay	2018–2021	46.71	−8.69		81	94.2	0.289 ± 0.016	0.296 ± 0.015	0.016 ± 0.018	18 (9)	807 (756)
	N. Portugal	2018–2021	41.53	−12.92		97	94.2	0.297 ± 0.016	0.305 ± 0.014	0.026 ± 0.019	27 (14)	986 (931)
Fjords	Osterfjorden	2018	60.62	5.52	60–170	156	100.0	0.330 ± 0.017	0.332 ± 0.015	0.020 ± 0.017	24 (12)	871 (821)
	Korsfjorden	2017	60.16	5.09	75–450	25	94.2	0.337 ± 0.017	0.333 ± 0.014	−0.007 ± 0.021	12 (1)	563 (523)
	Bjørnafjorden	2017–2018	60.12	5.62	350	210	100.0	0.328 ± 0.016	0.333 ± 0.014	0.020 ± 0.016	22 (14)	959 (907)
	Hardangerfjorden	2017–2018	60.03	5.93	657	40	95.9	0.336 ± 0.017	0.334 ± 0.014	0.001 ± 0.020	14 (6)	675 (637)
	Boknafjorden	2017–2018	59.20	5.61	90	89	99.2	0.332 ± 0.016	0.335 ± 0.014	0.017 ± 0.018	21 (10)	890 (841)
Mediterranean	Alborán	2018–2021	36.00	−3.96		24	42.1	0.111 ± 0.017	0.111 ± 0.015	0.015 ± 0.028	7 (4)	29 (21)
	Balears	2018–2021	38.50	2.50		51	52.1	0.125 ± 0.017	0.119 ± 0.015	−0.011 ± 0.022	6 (5)	63 (52)
	Ionian Sea^a^	2019	38.09	22.74	533–570	63	50.4	0.112 ± 0.016	0.113 ± 0.015	0.039 ± 0.024	12 (8)	60 (45)
	Aegean Sea	2019	37.60	23.26	319–370	60	52.1	0.124 ± 0.018	0.116 ± 0.015	−0.021 ± 0.020	7 (3)	46 (45)

^a^ Gulf of Corinth.

**FIGURE 2:**
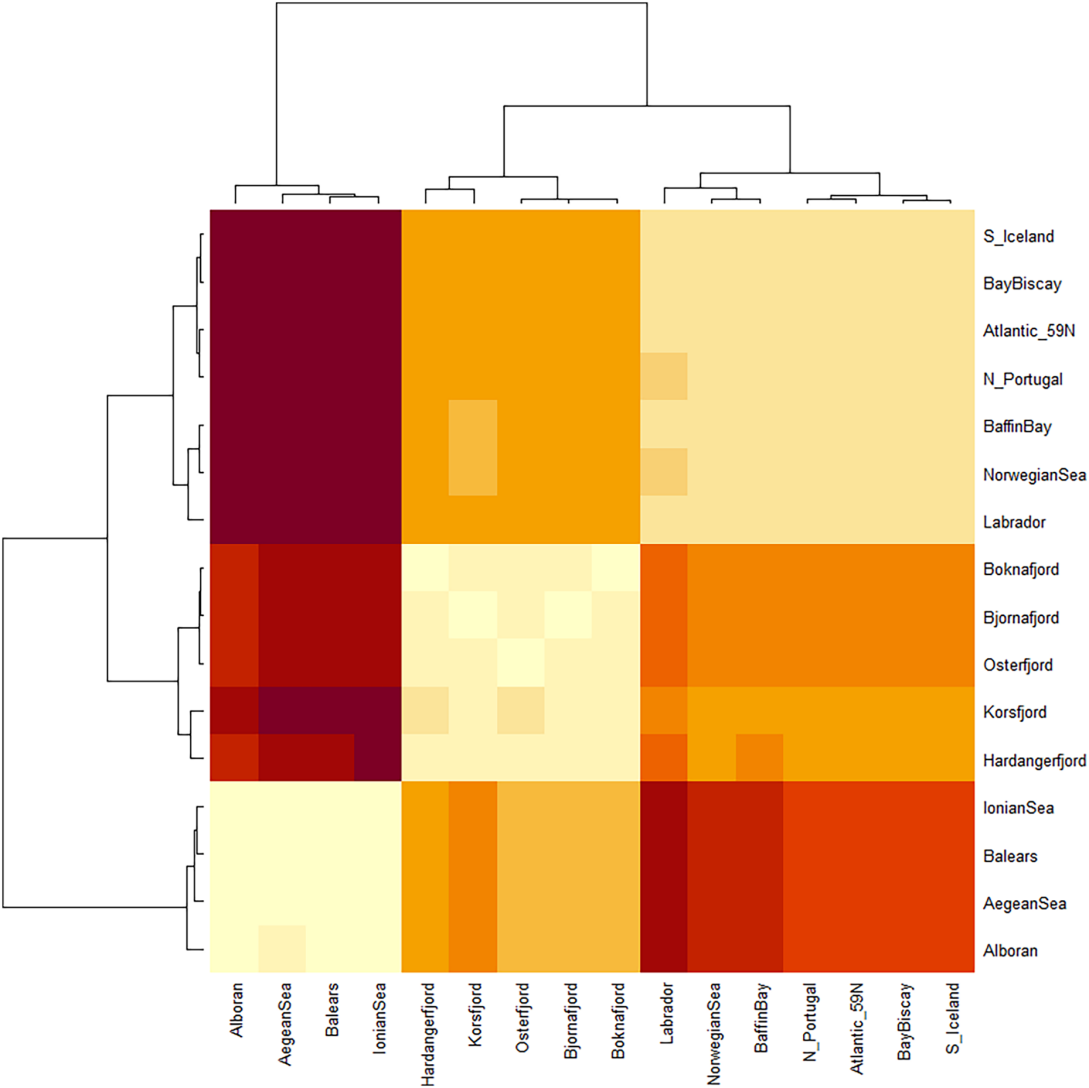
Heatmap *F*_ST_ coupled with dendrogram. Pairwise values and significance can be found in Table 2. Genetic differentiation creates three distinct groups: Atlantic open coast (i.e., “Ocean”), Norwegian fjords (i.e., “Fjords”), and “Mediterranean.” Colours depict the degree of differentiation: Beige colours indicate low differentiation and moving towards dark brown to indicate larger differentiation.

High levels of genetic differentiation were detected on a per‐locus basis, with half of the loci showing *F*_ST_ > 0.1. AMOVA revealed highly significant differentiation overall (*F*_ST_ = 0.186, *p* < 0.001), with 18.6% of the variation hosted among samples. The dendrogram coupled with pairwise *F*_ST_ revealed three distinct genetic groups (Figure 2): (i) samples from the Atlantic open ocean, hereafter called “Ocean,” (ii) samples from the Norwegian fjords, henceforth named “Fjords” for simplicity, and (iii) samples from the “Mediterranean.” AMOVA conducted using a hierarchical approach showed that the differentiation among Ocean, Fjord, and Mediterranean groups accounted for circa 25% of the total variation. Low but significant levels of genetic differentiation among samples within groups were also detected (*F*_SC_ = 0.004, *p* < 0.001). This was mostly driven by the Mediterranean samples, where small differences were registered in all pairwise Mediterranean comparisons (*F*_ST_ ranging from 0.012 to 0.076) except for the Western samples Alborán versus Balears (Table 2). Within the Fjords, Osterfjorden differed from all others with the exception of Korsfjorden, and significant differentiation was found between Boknafjorden and Bjørnafjorden. Most remarkable was the genetic homogeneity observed across the Ocean samples, covering both sides of the Atlantic and geographic distances up to 4000 km. The only exception within the Ocean samples was the genetic difference between the lanternfish from the Labrador and the Norwegian Sea (*F*_ST_ = 0.014, *p* = 0.014). In contrast to genetic homogeneity within each group, all pairwise comparisons concerning different groups were significant, with the largest degree of structure detected between the Ocean and Mediterranean components (*F*_ST_ ranging between 0.45 and 0.58) and the lowest between Ocean and Fjords (*F*_ST_ from 0.13 to 0.19).

**TABLE 2:** Genetic differentiation between geographically explicit samples estimated for the total 121 SNP loci: Heatmap of pairwise *F*_ST_ values in the bottom diagonal and corresponding *p*‐values after 10,000 permutations in the top diagonal, with the ones significantly different from zero after FDR correction highlighted in boldface type. Greener colours indicate low differentiation, increasing towards red to indicate larger differentiation.

		Ocean							Norwegian fjords					Mediterranean			
		Baffin Bay	Labrador	Norwegian Sea	S. Iceland	Atlantic 59 N	Bay Biscay	N. Portugal	Osterfjorden	Korsfjorden	Bjørnafjorden	Hardangerfjorden	Boknafjorden	Alborán	Balears	Ionian Sea	Aegean Sea
Ocean	Baffin Bay	—	0.739	0.371	0.978	1.000	0.943	0.934	**0.000**	**0.000**	**0.000**	**0.000**	**0.000**	**0.000**	**0.000**	**0.000**	**0.000**
	Labrador	0.000	—	**0.014**	0.355	1.000	0.136	0.129	**0.000**	**0.000**	**0.000**	**0.000**	**0.000**	**0.000**	**0.000**	**0.000**	**0.000**
	Norwegian Sea	0.000	0.014	—	0.958	1.000	1.000	1.000	**0.000**	**0.000**	**0.000**	**0.000**	**0.000**	**0.000**	**0.000**	**0.000**	**0.000**
	S. Iceland	0.000	0.000	0.000	—	0.411	0.188	0.036	**0.000**	**0.000**	**0.000**	**0.000**	**0.000**	**0.000**	**0.000**	**0.000**	**0.000**
	Atlantic_59N	0.000	0.000	0.000	0.000	—	0.589	0.244	**0.000**	**0.000**	**0.000**	**0.000**	**0.000**	**0.000**	**0.000**	**0.000**	**0.000**
	Bay Biscay	0.000	0.003	0.000	0.001	0.000	—	0.737	**0.000**	**0.000**	**0.000**	**0.000**	**0.000**	**0.000**	**0.000**	**0.000**	**0.000**
	N. Portugal	0.000	0.003	0.000	0.004	0.001	0.000	—	**0.000**	**0.000**	**0.000**	**0.000**	**0.000**	**0.000**	**0.000**	**0.000**	**0.000**
Fjords	Osterfjorden	0.154	0.173	0.136	0.160	0.165	0.151	0.146	—	0.062	**0.001**	**0.001**	**0.000**	**0.000**	**0.000**	**0.000**	**0.000**
	Korsfjorden	0.140	0.158	0.130	0.143	0.146	0.137	0.128	0.007	—	0.499	0.163	0.271	**0.000**	**0.000**	**0.000**	**0.000**
	Bjørnafjorden	0.144	0.162	0.132	0.149	0.155	0.143	0.138	0.006	0.000	—	0.087	**0.029**	**0.000**	**0.000**	**0.000**	**0.000**
	Hardangerfjorden	0.164	0.192	0.152	0.168	0.175	0.162	0.152	0.018	0.004	0.004	—	0.565	**0.000**	**0.000**	**0.000**	**0.000**
	Boknafjorden	0.148	0.170	0.135	0.151	0.162	0.145	0.139	0.011	0.001	0.004	0.000	—	**0.000**	**0.000**	**0.000**	**0.000**
Medit.	Alborán	0.514	0.565	0.517	0.472	0.458	0.465	0.448	0.226	0.297	0.219	0.250	0.216	—	0.524	**0.000**	**0.000**
	Balears	0.525	0.571	0.527	0.482	0.462	0.477	0.460	0.231	0.318	0.221	0.266	0.223	0.000	—	**0.000**	**0.000**
	Ionian Sea	0.534	0.583	0.538	0.487	0.461	0.482	0.463	0.234	0.337	0.225	0.277	0.233	0.046	0.025	—	**0.001**
	Aegean Sea	0.520	0.571	0.523	0.473	0.449	0.469	0.450	0.223	0.318	0.213	0.260	0.219	0.076	0.047	0.012	—

**FIGURE 3:**
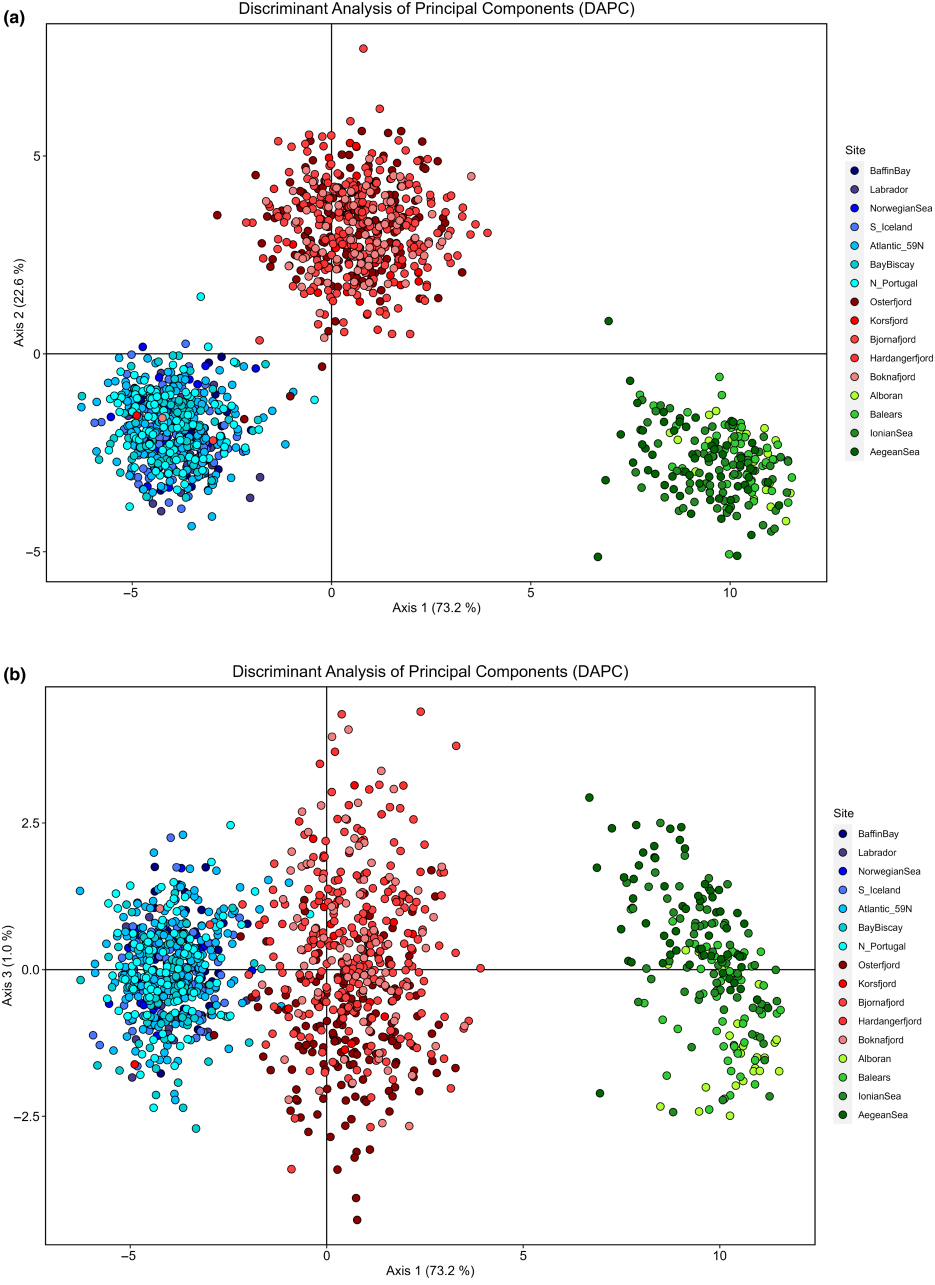
Genetic differentiation among *Benthosema glaciale* samples assessed with 121 SNP loci using Discriminant Analysis of Principal Components (DAPC) after retaining 110 principal components and 15 discriminant functions: (a) axis 1 and 2, and (b) axis 1 and 3. Individuals from different sampling sites are represented by coloured dots.

The DAPC based upon geographically‐explicit samples (Figure 3a) revealed large differences among the three groups (Ocean, Fjords, and Mediterranean). The first axis of differentiation (71% of the variation) showed that the relative position of these three groups was concordant with pairwise *F*_ST,_ whereas the second axis, accounting for 22.7% of the variation, further discriminated the Fjords from the remaining samples. However, bringing the third axis (1.5%) into play unravelled a gradient of differentiation within the Mediterranean samples (Figure 3b).

**FIGURE 4:**
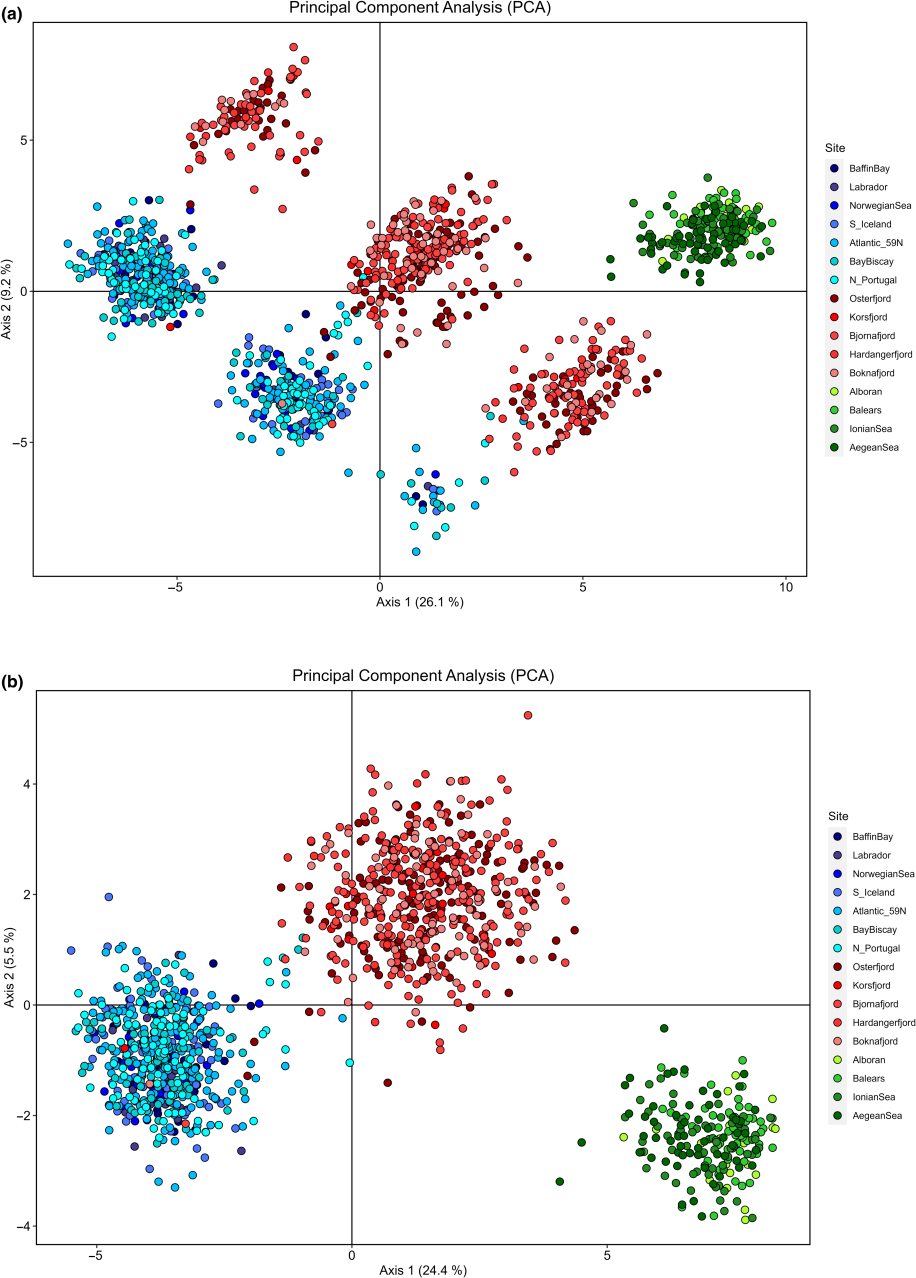
Principal Component Analysis biplot generated using the 121 polymorphic markers (a) and the 84 LD‐pruned ones (b). Blue dots depict ocean samples, red dots identify Fjords, and green dots represent Mediterranean samples. The distribution of individuals corresponding to the upper barplot into their putative karyotypes can be found in Figure S3a–c.

**FIGURE 5:**
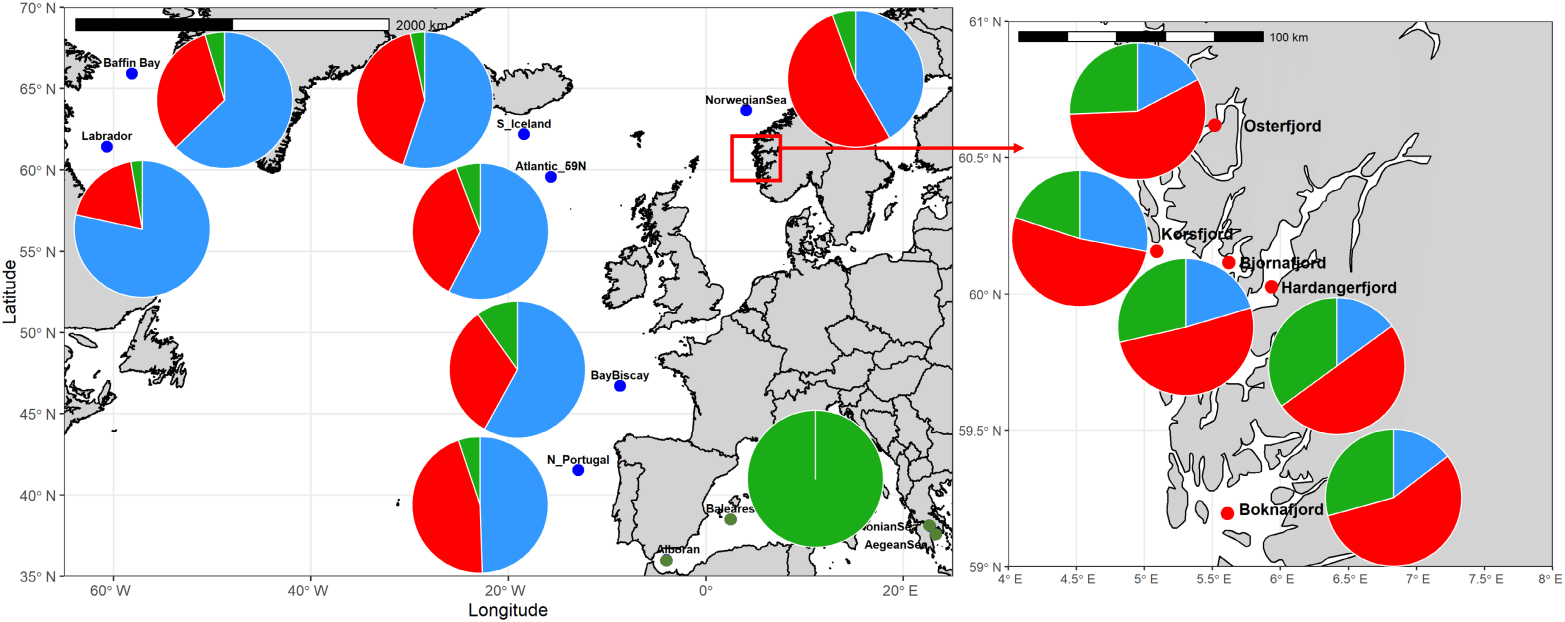
Frequency of occurrence of haplogroups per sample. One of the homokaryotypes (depicted in green) is fixed in the Mediterranean for the second most common arrangement of the Fjord samples, whereas the alternative homokaryotype (coloured in blue) is most frequent in the Oceanic samples.

The PCA biplot revealed three distinct clusters, one located at mid‐distance from the other two, that were modulated by geographic and/or habitat differentiation as ocean, fjords and Mediterranean fish clustered distinctly in the *Y*‐axis of each stripe (Figure 4a). The haplogroup in central position contained the individuals that are heterozygotes (AB) for those loci, i.e., heterokaryotypes (see Table 3), whereas the flanking haplogroups contained the AA and BB homozygotes (i.e., homokaryotypes), respectively (see Figure S3 for the individual distribution of karyotypes). Interestingly, the three variants were not evenly distributed, i.e., in the Mediterranean only BB is present, AA is more frequent in the Ocean samples (56.7%), whereas Fjord individuals seemed to better conform to Mendelian proportions (18.5%, 53.7%, and 27.9%, respectively). In two of the samples, the proportion of the AA component slightly deviated from the general pattern of the group, i.e., in Korsfjorden towards the Ocean profile, whereas in the Norwegian Sea towards the Fjord profile (Figure 5). The second axis of the PCA (9.2% of the variation) accounted for the geographic differentiation and placed the Mediterranean closer to the Fjords than to the Ocean samples, in agreement with pairwise *F*_ST_ (Table 2). The loadings on the first axis (26.1% of the variation) identified 12 loci, all of them linked (all pairwise Fisher's exact test *p*‐values < 0.0001) with IA = 5.42 (*p* = 0.001) and rd ranging between 0.16 and 0.92 with an average of 0.49 (*p* = 0.001) (Figure S4), which were responsible for the striation.

**TABLE 3:** Allele frequency per karyotype (AA, AB, BB) in the putative structural variant for individuals belonging to Fjord, Ocean, and total samples. Loci in boldface font globally contribute the most to the PCA haplogroup clustering and were used for haplotype reconstruction. The corresponding PCA plots can be found in Figure S3a–c. Greener colours indicate low differentiation, increasing towards red to indicate larger differentiation.

Locus	Fjords			Ocean			Total		
	AA	AB	BB	AA	AB	BB	AA	AB	BB
**OOFJ01129979.1_5655**	1.00	0.50	0.00	1.00	0.50	0.00	1.00	0.50	0.00
**OOFJ01080547.1_2856**	1.00	0.50	0.00	1.00	0.50	0.00	1.00	0.50	0.00
**OOFJ01103092.1_711**	1.00	0.50	0.00	1.00	0.50	0.00	1.00	0.50	0.00
**OOFJ01017723.1_4547**	1.00	0.50	0.00	1.00	0.50	0.00	1.00	0.50	0.00
**OOFJ01017507.1_9780**	1.00	0.51	0.01	1.00	0.60	0.18	1.00	0.55	0.02
OOFJ01018623.1_6494	0.99	0.44	0.00	1.00	0.28	0.00	1.00	0.37	0.08
OOFJ01103619.1_8660	0.99	0.48	0.00	0.61	0.31	0.00	0.70	0.41	0.00
OOFJ01039447.1_4050	0.99	0.48	0.00	0.65	0.31	0.00	0.73	0.41	0.00
OOFJ01018827.1_5831	0.96	0.52	0.00	0.68	0.43	0.00	0.74	0.48	0.04
OOFJ01002246.1_6178	0.96	0.45	0.00	0.68	0.29	0.00	0.74	0.38	0.00
OOFJ01079243.1_2636	0.96	0.45	0.00	0.68	0.28	0.00	0.74	0.38	0.02
OOFJ01102876.1_1855	0.97	0.43	0.00	0.42	0.10	0.00	0.55	0.28	0.00

**FIGURE 6:**
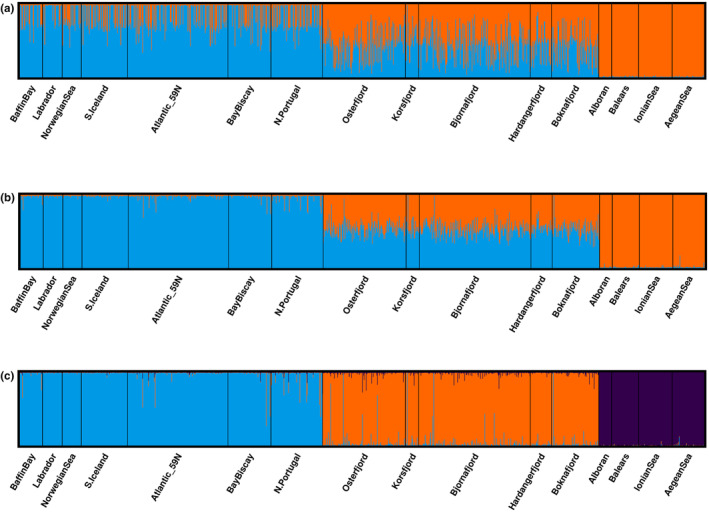
Barplot representing the proportion of individuals' ancestry to cluster at *K* = 2 as determined by the Evanno test after Bayesian clustering in structure assessed from (a) the set of 121 SNP loci and (b) the 84 LD pruned loci. Barplot (c) depicts the best solution for the pruned data set according to Puechmaille's statistics (*K* = 3) with samples coloured according to habitats (blue: Oceanic; red: Fjords; and green: Mediterranean).

The Evanno test strongly suggested *K* = 2 as the most likely number of genetic groups when using the total set of 121 markers (Δ*K* = 14,440, Figure S5). As STRUCTURE was conducted in an unsupervised manner, the individual ancestry to the clusters shown in the barplot using the total 121 loci reflected the outcome of PCA analysis (Figure 6a), thus showing a larger resemblance of the Mediterranean fish towards the Fjords than towards Ocean ones. On the other hand, two of Puechmaille's statistics suggested either *K* = 3, whereas the remaining ones suggested *K* = 4 and *K* = 5, respectively (Figure S5). The bar plots built using from three to five clusters showed reminiscences of the ancestry due to the three‐stripe pattern (Figure S6a–c).

### 3.2 Haplotype Groups

The clustering pattern for the oceanic and/or fjord samples was driven by 12 loci in strong linkage. However, adding the Mediterranean samples to the analysis not only changed the orientation of the clustering (see Figure S3) but also reduced to five the number of loci that showed a perfect frequency of 1–0.5–0 per haplogroup, respectively (see Table 3). Those five markers generated seven haplotypes: two dominating ones and five of barely testimonial presence (overall frequencies < 0.3%). The most abundant ones, Hap_2 and Hap_7 (overall frequency of 51.4% and 45.9%, respectively), were unevenly distributed across samples. In the Mediterranean only BB is present; AA is more frequent in the Ocean samples (56.7%) whereas Fjord individuals seemed to accommodate better to Mendelian proportions (18.5%, 53.7%, and 27.9%, respectively). In two of the samples, the proportion of the AA component slightly deviated from the general PCA pattern of the group, i.e., in Korsfjorden towards the Ocean profile, whereas in the Norwegian Sea towards the Fjord profile. The second axis of the PCA (9.2% of the variation) accounted for the geographic differentiation and placed the Mediterranean closer to the Fjords than to the Ocean samples, in agreement with pairwise *F*_ST_ (Table 2). Fjord samples displayed lower haplotype diversity than the Ocean ones, whereas in the Mediterranean barely one haplogroup was present (homokaryote BB).

Haplotype distribution also contributed to population structure. Thus, AMOVA based upon haplotypes revealed highly significant differentiation (*F*_ST_ = 0.29, *p* < 0.001), with 29.5% of the variation hosted among samples, whereas when using a hierarchical approach, the variation hosted among groups was larger (38.2%), but no significant differentiation was detected within groups. Pairwise *F*_ST_ for haplotypes revealed null differentiation within groups (except again for Labrador vs. Norwegian Sea) and strong differentiation between groups (Table S1). The levels of differentiation between Kosfjorden and the Mediterranean samples further deviate from the general Fjord pattern and resemble the Ocean one.

### 3.3 LD‐Pruned Analyses

Fisher exact tests were used to heavily LD‐prune the dataset, thus retaining 84 markers on which the former battery of genetic structure analyses was again deployed. Broadly speaking, two main types of results were obtained with the LD‐pruned dataset. The outcome from unsupervised analyses such as PCA or STRUCTURE was strongly modified as the striation disappeared from the PCA (Figure 4b), leaving only geography or habitat differentiation as the driver discriminating the three distinct clusters of individuals. The PCA first axis of differentiation (24.4% of the variation) separated Mediterranean, Fjord, and Ocean samples, whereas the second axis (5.5%) further distinguished the Mediterranean from the Fjords. Likewise, structure barplots revealed radically different results using LD‐pruned loci. Again, the Evanno test suggested *K* = 2 as the most likely solution (Δ*K* = 30,160, Figure S7) but yielded two very distinct and relatively homogenous groups: the Ocean samples on one side and the Mediterranean on the other one, with the Fjords in an intermediate position (Figure 6b). Puechmaille's statistics suggested *K* = 4 or *K* = 6 (see Figures S7 and S8b, a‐c), whereas the most likely number of clusters based upon the information provided by PCA would be *K* = 3 (Figure 6c), also in agreement with the Evanno test's second option. Furthermore, LD‐pruned loci unravelled the existence of five putative Ocean migrants within the Fjord samples (one in Bjørnefjord, Boknafjorden, and Korsfjorden, and two in Osterfjorden; see Figures 4b and 6b,c). Four of these putative migrants belonged to the heterokaryogroup AB, showing haplotypes 2 and 7, whereas the one found in Kosfjord was a homozygote AA (hap2, hap2). It is worth noting that Korsfjorden is the most open of the fjords sampled within the present study, and the genetic profile in this location is slightly closer to the ocean samples (see Figure 5).

In contrast, the supervised analyses based upon a priori grouping of the individuals using geographically‐explicit locations mirrored the outcome using the full dataset of 121 loci. This was the case of genetic diversity (Table S2), pairwise *F*_ST_ (Table S3), and DAPC analyses (Figure S9a,b). In the AMOVA, the overall differentiation was slightly larger for the LD‐pruned dataset (*F*_ST_ = 0.197, *p* < 0.001), with 19.7% of the variation hosted among samples, whereas 24.8% of the variation was found among groups (Fjords, Ocean, Mediterranean). Interestingly, in the LD‐pruned dataset, Fjord samples displayed similar levels of differentiation from the Ocean ones and from the Mediterranean (Table S3), in contrast with the non‐pruned dataset in which Fjords were genetically closer to the Ocean samples than to the Mediterranean.

## 4 Discussion

Sustainable fisheries management requires, among others, detailed knowledge on the number and distribution of the isolated or semi‐isolated populations of a species—information that is lacking for most mesopelagic fishes. The present study fills the knowledge gap on population genetic structure of a prospective fishing resource, the glacier lanternfish, across most of its distribution range. A suite of 84 LD‐pruned SNPs revealed three highly distinct populations inhabiting the Mediterranean Seas, Norwegian Fjords, and both sides of the Atlantic Ocean, respectively, with very weak differentiation within them. This spatial structure, with sharp genetic subdivisions, would not be expected if random genetic drift was predominantly responsible for the genetic differentiation between these lineages (Meyer et al. 2024). The remarkably high level of connectivity detected across the North Atlantic contrasts with the low motility of the species and would invoke passive dispersal in the absence of current barriers to gene‐flow as the main explanatory driver. However, demographic history plays also a role, as different stocks might have been isolated during the glaciation due to the ice front and lower water level.

### 4.1 Genetic Population Structure

Ocean samples collected across the Atlantic in a geographic span of 300–4000 km displayed no genetic differentiation, except for the comparison Labrador versus Norwegian Sea. This limited differentiation could be attributed to the homogenising effect that drifting poses on a species inhabiting a zone lacking barriers to dispersal. Mesopelagic fishes seem to prefer spawning and nursery areas in frontal and upwelling zones to the areas in central waters and take advantage of the current and counter‐current to transport their eggs and larvae up to 1000 km (see Cornejo and Koppelmann (2006) and references therein). Dispersal mediated by planktonic larvae has also been claimed to the responsible for the lack of genetic structure reported across vast oceanic areas for other myctophid species (Kojima, Moku, and Kawaguchi 2009; Van de Putte et al. 2012). The number of micro‐increments in the larval zone in otoliths of *B. glaciale* suggests that the larval period of this species lasts around 42 days (García‐Seoane, Meneses, and Silva 2014), which could provide enough time of passive transport to ensure connectivity. In addition to early drift, older stages of lanternfish can become expatriated by ocean currents into both sides of the Atlantic. Similarly, they can also be advected into waters at higher latitudes where they are lost from the reproductive population (Saunders et al. 2017). In our study, the only pair of Ocean sample sets that displayed significant structure was Labrador versus Norwegian Sea. We hypothesize that a combination of the ascending warm Norwegian current along the Norwegian coast and the descending cold E. Greenland current obstructs the connectivity between these geographically distant samples. Similarly, in the southern hemisphere, microsatellites revealed that the myctophid *Electrona antarctica* displays very high genetic diversity coupled with a striking lack of genetic differentiation on a circumantarctic scale, thus underscoring the large‐scale homogenizing effect of the Southern Coastal Current in the Southern Ocean (Van de Putte et al. 2012). In this geographic area, gene flow mediated by larval dispersal accounts for the weak/absent genetic structure observed on, e.g., humped rockcod *Gobionotothen gibberifrons* (Matschiner, Hanel, and Salzburger 2009) and Antarctic silverfish *Pleuragramma antarctica* (Zane et al. 2006). The permeability of physical barriers to dispersal and gene flow in the mesopelagic zone, which is continuous in the North Atlantic, strongly shapes the modes of differentiation in the taxonomically diverse lanternfish family. Thus, modes of speciation relying exclusively on geographical separation are less relevant than those relying on evolution of assortative mating through divergent habitat use and/or sexual signals, including visual signals from bioluminescent light organs (Freer et al. 2022).

Unlike open waters, the hydrological and topographic characteristics of fjords may create unique habitat conditions (Farmer and Freeland 1983). In Norway, the sills typically present at the mouths of the fjords hinder deep‐water exchange with the adjoining coastal areas and may act as a physical barrier limiting gene flow. Thus, Norwegian fjords with sill depths exceeding 130 m resulted in genetically homogeneous populations of *B. glaciale*, whereas a significant genetic divergence was detected when sill depths were shallower (75 m) (Kristoffersen and Salvanes 2009). The shallow continental shelf and behaviourally imposed deep distribution limit the exchange of adults among fjords (Kristoffersen and Salvanes 2009), whereas pelagic early life stages of *B. glaciale*, which typically are found much closer to the surface (Olivar et al. 2014), suggest greater dispersal abilities, and hence population differentiation at micro‐geographic scale is less likely. However, in our study, the 40% of pairwise Fjord comparisons revealing significant structure collided with the absolute lack of differentiation described by Kristoffersen and Salvanes (2009), whereas for Suneetha and Salvanes (2001), genetic homogeneity was detected in fjords with sill depths exceeding 130 m, both studies using allozymes. Here, Osterfjorden, the northernmost of the analysed fjords, differed from all the remaining fjords but was the geographically closest one. This differentiation could be attributed to the shallow depth of Ostefjord's sill hampering connectivity, in agreement with the significant divergence reported for shallow fjords (Suneetha and Salvanes 2001). In addition to that, the only pairwise comparison that yielded weak yet significant structure was Boknafjorden versus Bjørnafjorden, and although both have deep sills, the complex configuration of the coastline could aid in promoting such differentiation. Suneetha and Salvanes (2001) also pointed at limited movement of adults between fjords as inferred from differences in growth and age composition, thus suggesting that connectivity among fjords is more likely to happen during the drifting pelagic eggs and larvae stages, as it has been also suggested in the open coast (Van de Putte et al. 2012).

The large differentiation between Fjord and Ocean *B. glaciale* reported in our study is in agreement with what former studies revealed using allozymes (Suneetha and Salvanes 2001; Kristoffersen and Salvanes 2009) and could be attributed to the barrier that the continental shelf represents for the exchange of genetic material between fjords and open waters. Interestingly, the fjord sample showing the lowest genetic differentiation with the Ocean samples (both in terms of pairwise *F*_ST_ of all markers as well as haplotype frequencies) came from Kosfjorden, a fjord with a very deep sill (450 m). The fjord versus off‐shore differentiation has also been described in other species with high dispersal capacity, such as, e.g., tunicate (*Ciona intestinalis*) (Johannesson et al. 2018), northern shrimp (Hansen et al. 2021), European sprat (Quintela et al. 2020), Atlantic cod (Ruzzante et al. 1997; Westgaard and Fevolden 2007; Pampoulie et al. 2011), or haddock (*Melanogrammus aeglefinus*) (Berg et al. 2021).

Past isolation followed by secondary contact can also result in discreet clusters with potentially different habitat usage (Bierne et al. 2011). The standard genetic models of glacial refugia and colonization predict lower genetic diversity in formerly glaciated areas and higher genetic diversity in glacial refugia (Hewitt 1996); prediction based on the idea that genetic diversity is lost during migration as not all genotypes migrate. However, this prediction may not hold in areas with secondary contact, where admixture of formerly separated lineages rather increases genetic diversity (Petit et al. 2003), particularly in species with high dispersal capacity. Thus, an excess of linkage disequilibrium and of genetic diversity at the suture zone is a unique signature of secondary contact (Bertl, Ringbauer, and Blum 2018). Although the current dataset does not allow for empirically testing demographic models, both the largest genetic diversity and linkage disequilibrium per sample were detected in the fjords, i.e. formerly glaciated areas (see figure 1 in Jenkins, Castilho, and Stevens (2018)). Therefore, the fjords could be the contact point between populations flowing from two different refugia, the Atlantic Ocean and the Mediterranean. A number of fish species are known to have had glacial refugia in the Northern Atlantic (Gysels et al. 2004; Le Moan, Gagnaire, and Bonhomme 2016; Cayuela et al. 2020; Benestan et al. 2021), whereas the Mediterranean is known to be one of the seven potential LGM marine glacial refugia based on marine and coastal terrestrial evidence (see Maggs et al. 2008). Another example of secondary contact scenarios are the population breaks detected between the Atlantic and the Mediterranean for a number of species such as European flat oyster (*Ostrea edulis*) (Lapègue et al. 2023), European lobster (*Homarus gammarus*) (Jenkins et al. 2019), seahorses (Riquet et al. 2019; Meyer et al. 2024), European sea bass (*Dicentrarchus labrax*) (Tine et al. 2014), or harbor porpoise (*Phocoena phocoena*) (Fontaine 2016).

In contrast to the patterns observed in the Atlantic Ocean, genetic connectivity in the Mediterranean Sea is strongly shaped by oceanographic processes such as current patterns and oceanographic discontinuities (Galarza, Carreras‐Carbonell, et al. 2009; Schunter et al. 2011). On the Spanish coast, the entry of less saline Atlantic waters through the shallow Strait of Gibraltar represents a barrier to gene flow for numerous species (Galarza, Carreras‐Carbonell, et al. 2009; Galarza, Turner, 2009; Marie et al. 2016) and could account for the mesopelagic fish diversity being much lower in the Mediterranean than in the adjacent Atlantic waters (Olivar et al. 2022). In particular, the Almería‐Oran Front (AOF) is the main point of genetic break between the Atlantic Ocean and the Mediterranean (Patarnello, Volckaert, and Castilho 2007). In a comprehensive review, Pascual et al. (2017) concluded that, although genetic differentiation can happen independently of the presence of a front, oceanographic fronts do reduce gene flow in highly mobile species with a larval stage longer than 2–4 weeks, whereas benthic species and/or with larval phase < 2 weeks have more significant genetic breaks between localities. The Mediterranean *B. glaciale* samples analysed here displayed very weak, albeit significant, differentiation in a longitudinal gradient towards the Greek Seas, which would challenge the expectations derived by the duration of the larval period in this species, even if taking into consideration that in the warm Mediterranean waters larval period could be expected to be shorter than 42 days; however, the influence of drift in later stages of life cannot be dismissed either (Saunders et al. 2017). The westernmost sample (Alborán), which was collected in the area of AOF, did not differ from the one in Balears. However, along the Greek coastline, the Ionian and Aegean Seas shape a complex ecosystem combining a highly irregular coastline and semi‐isolated deep basins where differentiation has been formerly attributed to a combination of historic demographic processes as well as hydrological and ecological traits (see Sarropoulou et al. 2022 and references therein), which also leads to local differences in species composition (Somarakis, Isari, and Machias 2011; Kapelonis et al. 2023). The extremely weak, although significant, differentiation registered in the Greek samples analysed here does not conflict with the lack of structure detected in *B. glaciale* in the same area using mitochondrial markers (Sarropoulou et al. 2022), as the information provided by nuclear and mitochondrial DNA relates to different temporal scales.

The partition between the Atlantic and Mediterranean samples agrees with former studies conducted on a diverse array of marine taxa, such as sponges (Riesgo et al. 2019), molluscs (Pérez‐Losada et al. 2002; Lapègue et al. 2023), crustaceans (Reuschel, Cuesta, and Schubart 2010; Jenkins et al. 2019), or fish (Bargelloni et al. 2003; Quintela et al. 2020). The most attributed cause to this partition is the restricted gene flow between the Atlantic and Mediterranean basins, most frequently due to IBD and/or an oceanographic barrier to connectivity. However, one question that remains open is why the differentiation Mediterranean versus Atlantic Ocean is strikingly larger than Mediterranean versus Norwegian fjords and whether this potentially reflects some sort of local adaptation to the calmer waters of the partially enclosed body waters.

### 4.2 Candidate Structural Variants

The set of 121 polymorphic SNP markers used here conveyed different layers of information unravelled by different analyses. The picture provided by supervised analyses (AMOVA, pairwise *F*_ST_, DAPC) revealed a strong differentiation among habitats (Ocean, Fjords, and Mediterranean) inhabited by three distinct populations. The outcome of the supervised analyses was not affected by LD‐pruning the dataset, unlike what happened to the unsupervised analyses. On the other side, unsupervised methods (PCA, structure without priors) highlighted the effect of a subset of some 10% of the total loci, all in linkage disequilibrium, and some of them showed indications of being under positive selection by one or more outlier detection methods. Loadings on the first axis demonstrated that these loci were the main drivers of the three‐cluster pattern detected via the PCA biplot, as well as of the distinct membership to cluster reflected through structure. The maintenance of local adaptation in the face of gene flow is possible through three mechanisms: (a) linkage with divergent loci, (b) increased resistance to gene flow following secondary contact due to genetic incompatibilities, and (c) competition among genomic architectures, including mechanisms that reduce or suppress recombination (Tigano and Friesen 2016). In the last few years, molecular ecologists have paid increasing attention to the third mechanism and have shown that chromosomal rearrangements, which involve structural changes such as inversions, fusions, fissions, and translocations, may be a critical driver of local adaptation (Wellenreuther and Bernatchez 2018; Faria et al. 2019; Wellenreuther et al. 2019). Chromosomal inversions, reduce recombination, increasing linkage disequilibrium among loci at a potentially massive scale. Furthermore, the low recombination within chromosomal rearrangements may lead to independent evolution of the affected genomic regions despite high gene flow in the rest of the genome (Faria and Navarro 2010; Wellenreuther et al. 2019), which in turn allows the expression of specialized phenotypes associated with local adaptation (Berg et al. 2017; Mérot et al. 2018; Westram et al. 2018; Wellband et al. 2019).

The utility of PCA for the detection and characterization of inversions using high‐density SNP genotype data was developed by Ma and Amos (2012) based upon the rationale that if recombination is suppressed between inverted and non‐inverted segments, these two segments of different orientations represent two distinct lineages that have been diverging for many generations and accumulating mutations independently, and therefore detectable through PCA. The heterozygous individuals can be viewed as a perfect 1∶1 admixture of the two types of inversion homozygous populations, resulting in a special pattern consisting of three equidistant haplogroups. The validity of this approach has been demonstrated on different species genotyped using large SNP arrays, e.g., fruit fly (*Drosophila melanogaster*) (Nowling, Manke, and Emrich 2020), seaweed fly (*Coelopa frigida*) (Mérot et al. 2021), Arctic charr (*Salvelinus alpinus*) (Hale, Campbell, and McKinney 2021), or lesser sandeel (*Ammodytes marinus*) (Jiménez‐Mena et al. 2020). However, very modest SNP sets, such as the current one or the similarly sized one used on polar cod (*Boreogadus saida*) (Quintela et al. 2021), might also provide insights on putative chromosome inversions through PCA biplots. However, although PCA has been proposed as a method for discovering inversions (Ma and Amos 2012), long haplotypes under balancing selection or simply regions of reduced recombination (Lotterhos 2019) can produce signals that are indistinguishable from the signal left by inversions, and therefore, data based upon resequencing and long reads will be needed in glacier lanternfish to clarify the nature of this putative rearrangement and identify the genes involved in it.

Large‐scale inversions involved in ecological adaptation have been identified in Atlantic herring (*Clupea harengus*) in connection with temperature at spawning (Pettersson et al. 2019). For this species, high temperatures are a major stressor, particularly to southernmost populations that are frequently exposed to such conditions. In Atlantic cod, chromosome inversions underlie four supergenes (i.e., genomic regions containing sets of tightly linked loci that control multi‐trait phenotypic polymorphisms under balancing selection; Thompson and Jiggins 2014) allegedly linked to migratory lifestyle and environmental adaptations such as salinity tolerance (Matschiner et al. 2022). Inversions in chromosomes 2, 7, and 12 have been identified in coastal versus offshore samples of Atlantic cod (Sodeland et al. 2016; Johansen et al. 2020), whereas chromosome 2 has been shown to be highly divergent between spring and winter spawners within the Gulf of Maine (Barney et al. 2017). In the seaweed fly, the largest inversion Cf‐Inv(1) was associated with body size and covaried at a fine geographic scale with wrackbed habitat characteristics (Mérot et al. 2021). Likewise, East African mountain populations of the honeybee (*Apis mellifera*) highly diverged from neighbouring lowland populations at two extended regions in the genome, despite high similarity in the rest of the genome, suggesting candidate inversions governing local adaptation (Christmas et al. 2019).

In our study, the PCA also revealed strong genetic structure among groups: only one homokaryotype was present in the Mediterranean, whereas the alternative one was more frequent in the Ocean samples. In the Fjords, however, the proportions of homo‐ and heterokaryotypes approached Mendelian expectations. Haplotype 7, which was the only one present in the Mediterranean, was more abundant in the Fjord samples (overall 54%) than in the Ocean ones (20%), thus potentially suggesting that confined and partially enclosed water bodies present special conditions that, despite geographic distance, could eventually lead to some kind of convergent local adaptation. The age of the SVs is often older than the age of the contemporary populations in which they are studied, suggesting that their adaptive potential often relies on ancient polymorphisms (Marques et al. 2018; Wellenreuther and Bernatchez 2018), representing a source of standing variation for population divergence and adaptation. Ancient SVs have promoted the repeated evolution of ecotypes following the post‐glacial recolonization of new environments, as described in systems undergoing parallel evolution (Jones et al. 2012; Nelson and Cresko 2018; Morales et al. 2019).

### 4.3 Management Implications

Although the exact abundance and biomass of mesopelagic fishes is strongly debated (Pauly et al. 2021), high biomass estimates have sparked recent interests in developing potential fisheries to harvest these relatively intact fish stocks. However, before establishing any fishery on mesopelagic fish, several aspects of the biology of the target species, such as the number of populations and thus the number of management units, must be taken into consideration. Our finding of three distinct genetic populations of *B. glaciale* in the North Atlantic‐Mediterranean provides immediate relevance for the management of this species and may suggest that demographic properties should be mapped according to genetic population before attempting to set potential harvest levels. The data show that the ridge between the UK and Iceland that separates the North Atlantic proper from the Nordic Seas does not constitute a barrier to gene flow, thus suggesting that the surprisingly large differences (Melle et al. 2020) between these areas in terms of biomass levels and taxonomic diversity of mesopelagic micronekton (Klevjer, Melle, et al. 2020) are related either to environmental or ecological interactions. Even if the genetic data suggest strong inter‐area connectivity, delineation of the species into management units will need to incorporate these environmental or ecological interactions to meet pre‐requisites and establish a sustainable mesopelagic fishery.

## Conflicts of Interest

The authors declare no conflicts of interest.

## Supporting information


Data S1

## Data Availability

Information about the suite of SNP loci used as well as individual genotypes used in this study can be publicly accessed from the electronic archive of the Institute of Marine Research at https://hdl.handle.net/11250/3115642.
