# Supplementary material for: Genetics in the Ocean's Twilight Zone: Population Structure of the Glacier Lanternfish Across Its Distribution Range
Source: Evol Appl. 2024 Nov 6;17(11):e70032. doi: 10.1111/eva.70032 (PMC11540841; doi:10.1111/eva.70032)
Supplement: Supplementary file 1 — Data S1 [file EVA-17-e70032-s001.docx]

**SUPPLEMENT**

**Genetics in the ocean’s twilight zone: Population structure of the glacier lanternfish across its distribution range**

| **a)**  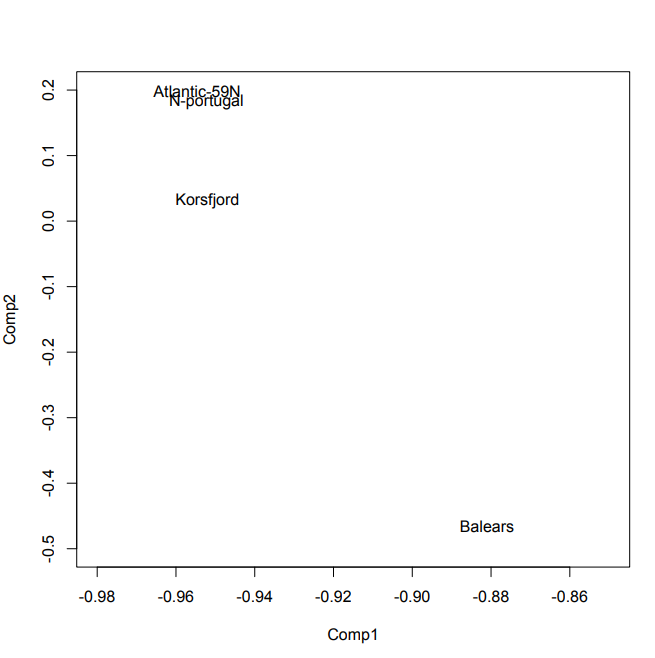 | |
| --- | --- |
| **b)**  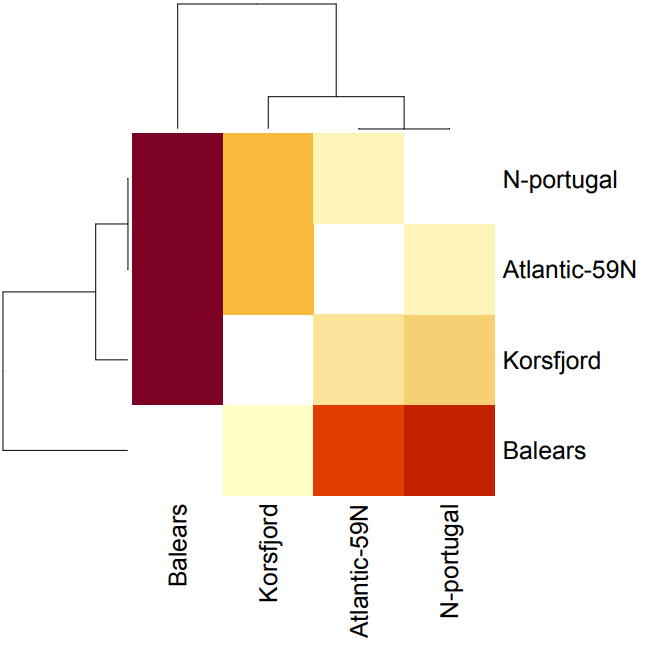 | **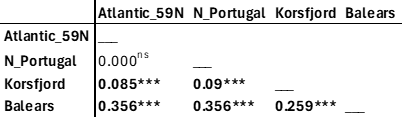** |

**Fig. S1.** Poolseq data for the four sites selected for SNP mining: Principal Component Analysis (a), and pairwise *F*_ST_: heatmap coupled with dendrogram together with *F*_ST_ values and significance (b).

^ns^(not significant), *** (P<0.001).


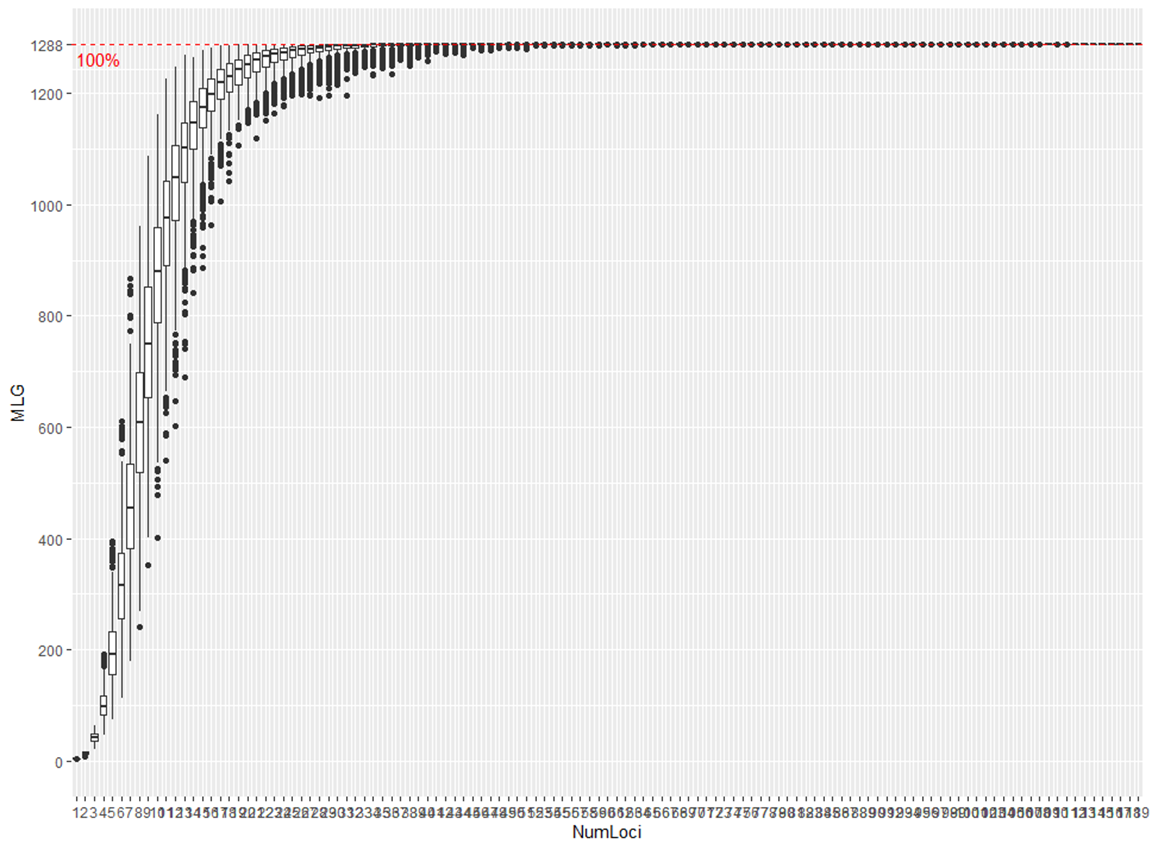


**Fig. S2.** Genotype accumulation curve calculated for the set of 121 polymorphic SNP loci using the total 1288 individuals.

| a)  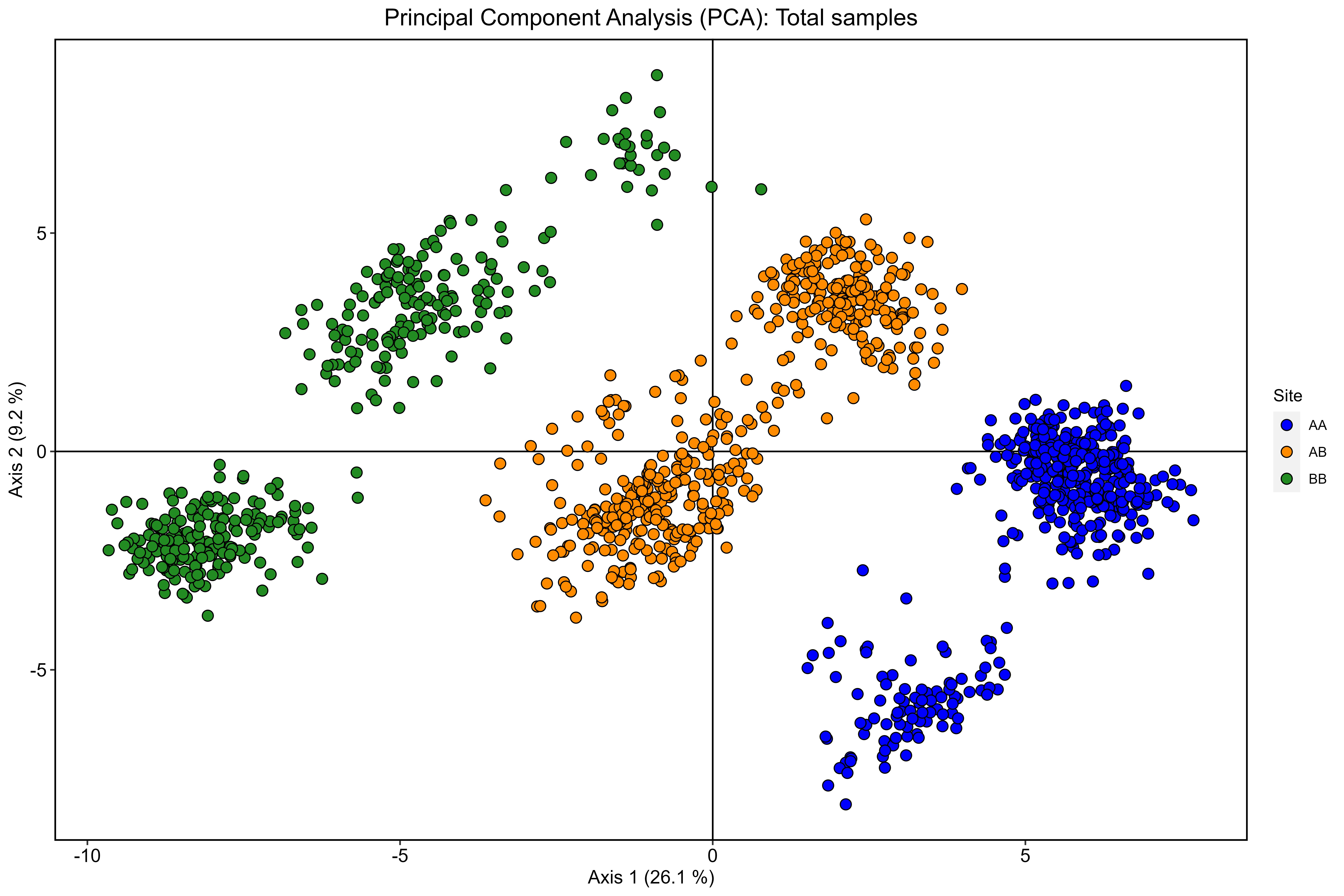 |
| --- |
| b)  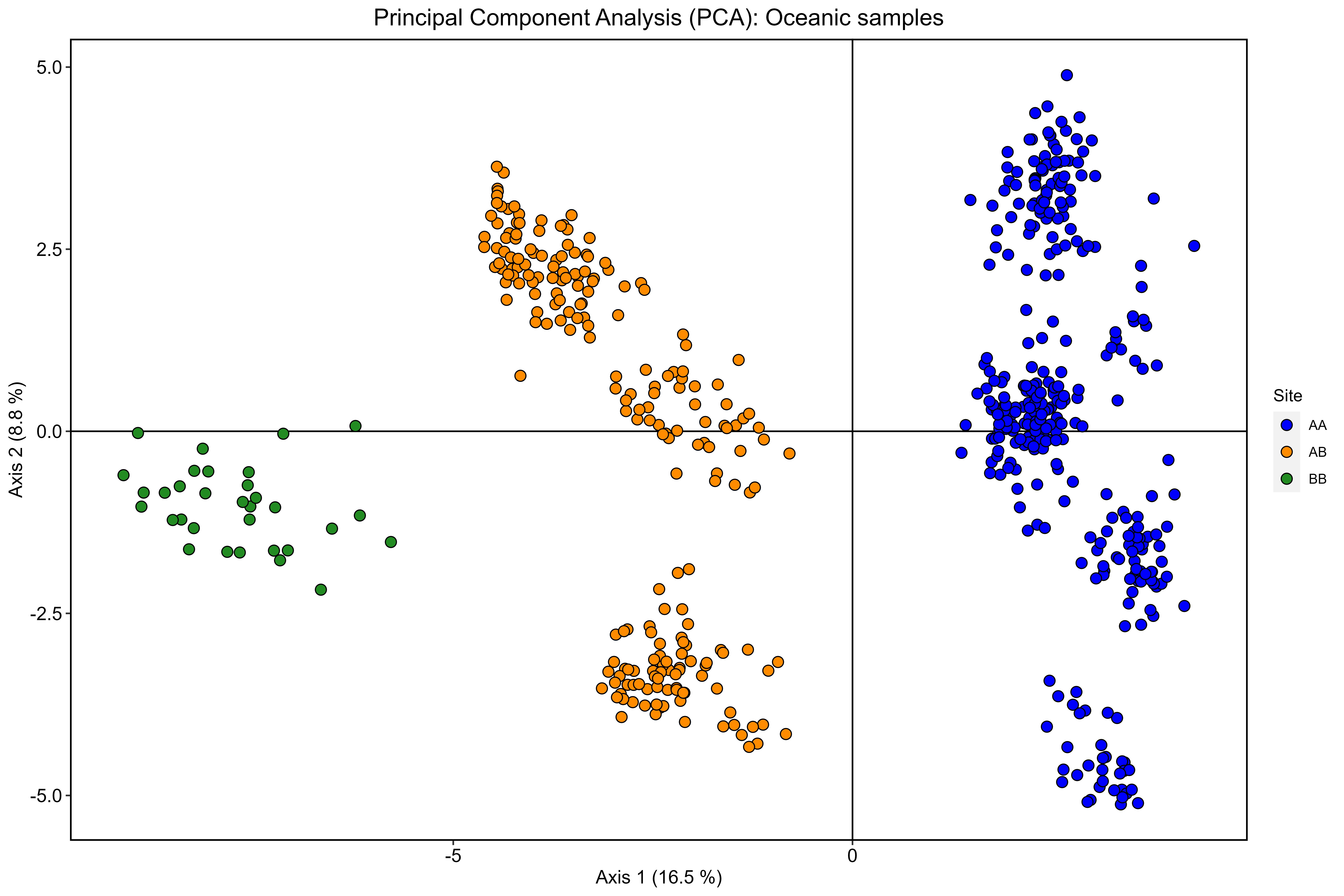 |
| c)  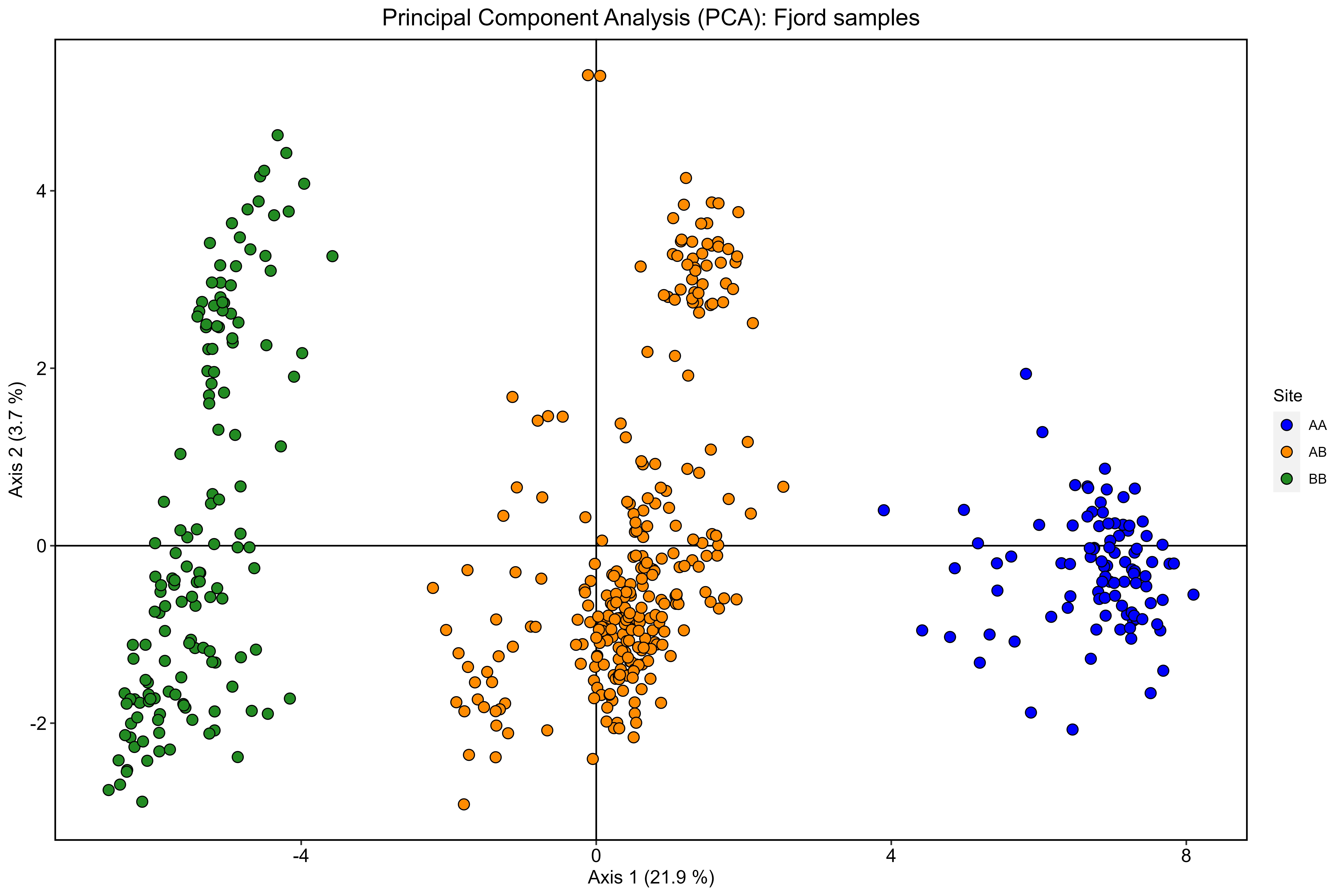 |

**Fig. S3.** Principal Component Analysis (PCA) of *Benthosema glaciale* genotyped at 121 loci: distribution of SV at a) all samples, b) oceanic samples and c) fjord samples.

| a)  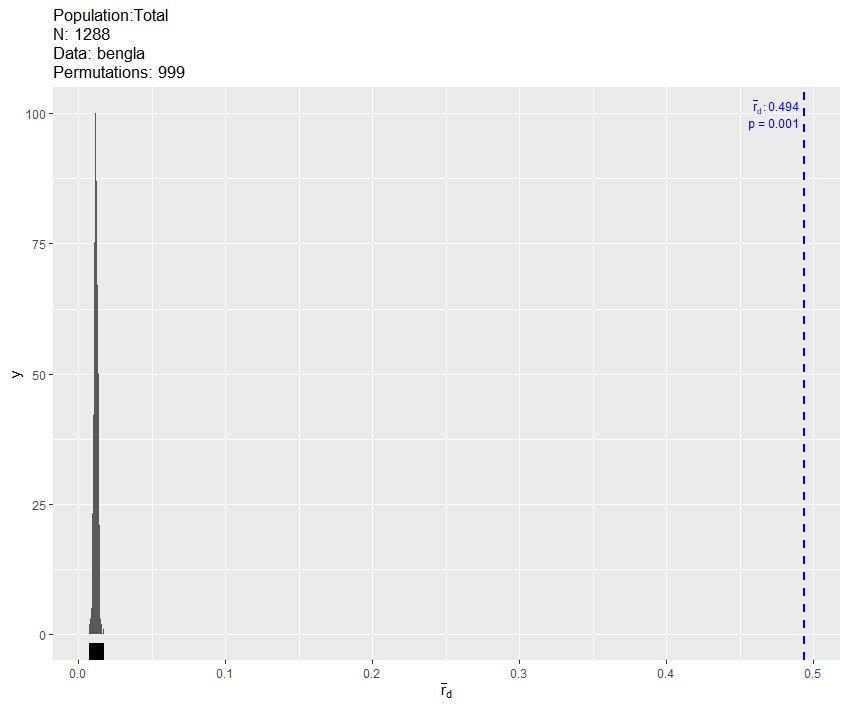 | b)  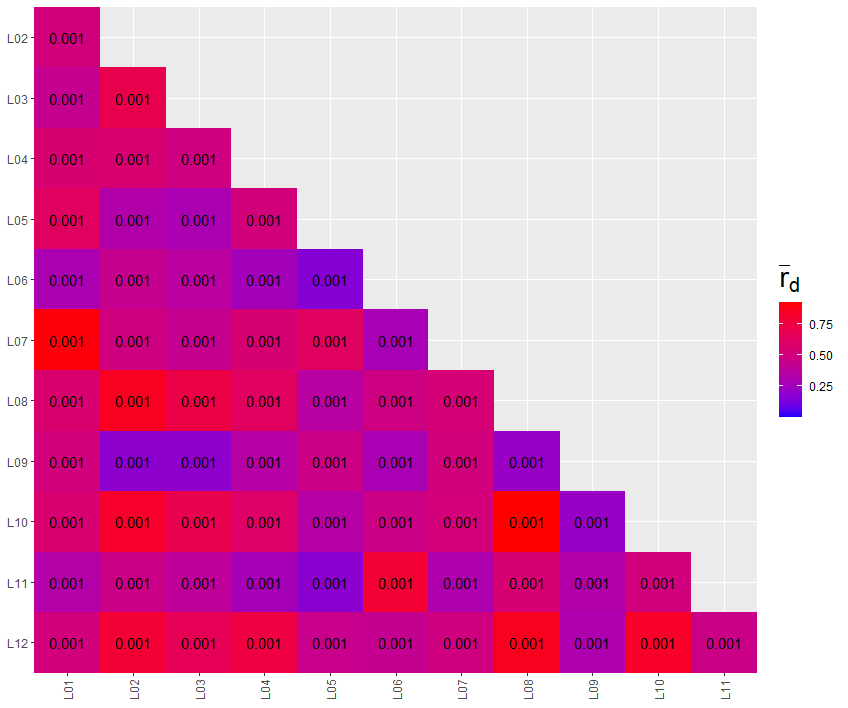 |
| --- | --- |

**Fig. S4.** Standardized index of association, rd (a) as the measure of multilocus genotypic linkage disequilibrium (LD) for the twelve SNPs (L01-L12) behind the haplogroups. The observed rd falls outside of the distribution expected under free recombination (dotted blue line) (b) Heatmap pairwise matrix of rd with P<0.01 indicating signiﬁcant LD (at 99.9% level). N, number of unique haplotypes


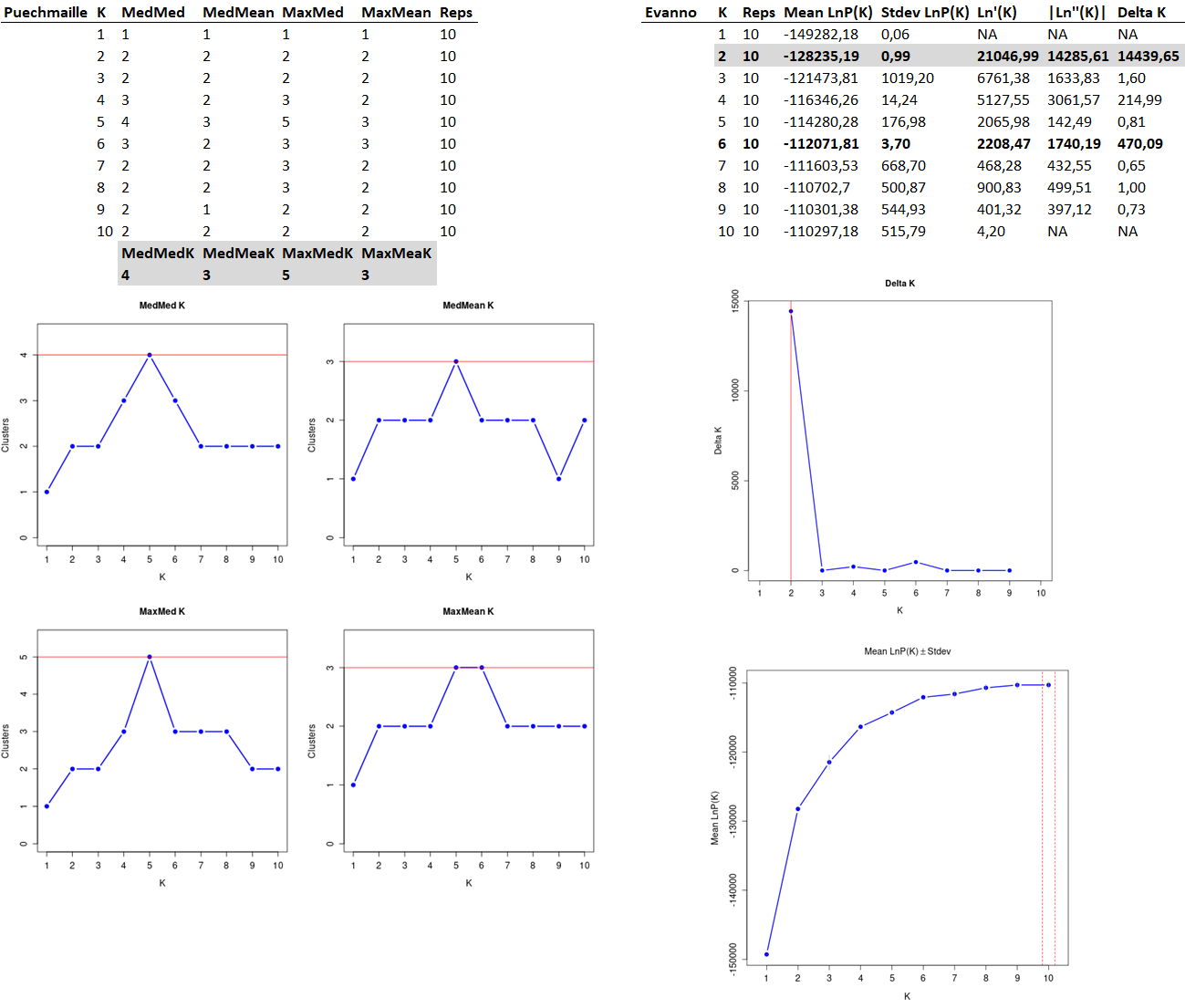


**Fig. S5.** *A posteriori* analysis of STRUCTURE outcome for the set of 121 loci following Puechmaille and Evanno’s statistics.

| a)  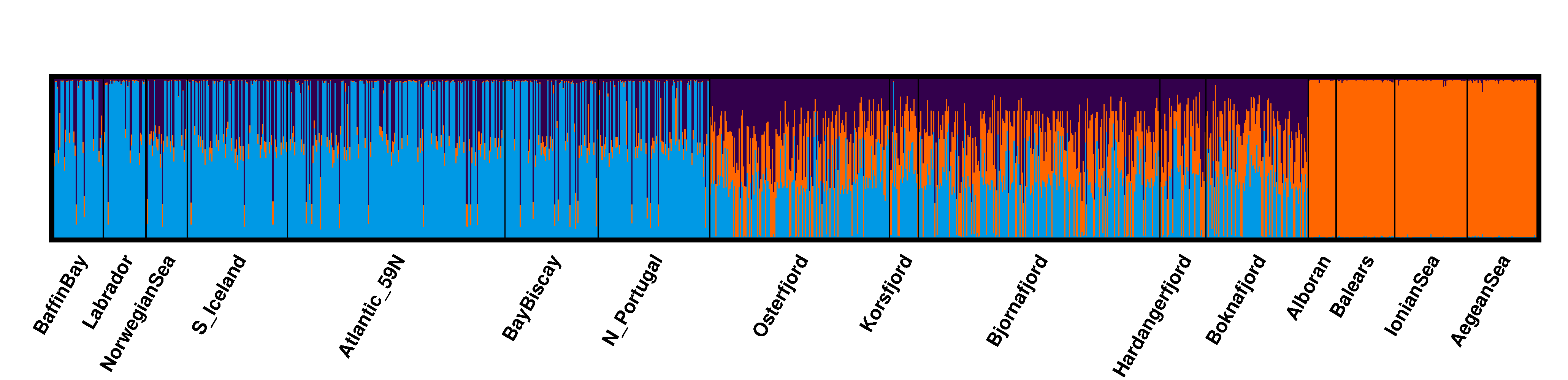 |
| --- |
| b)  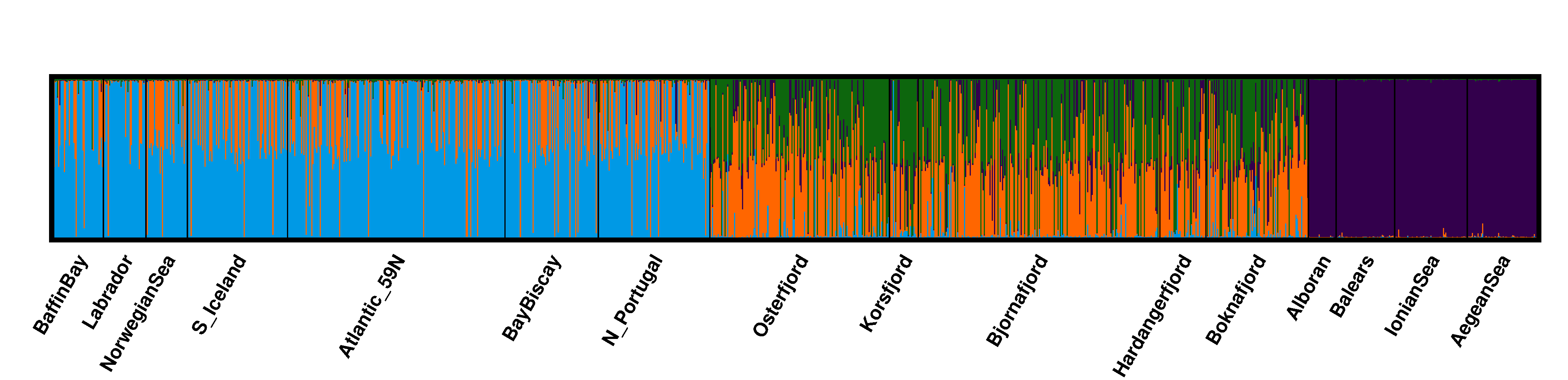 |
| c)  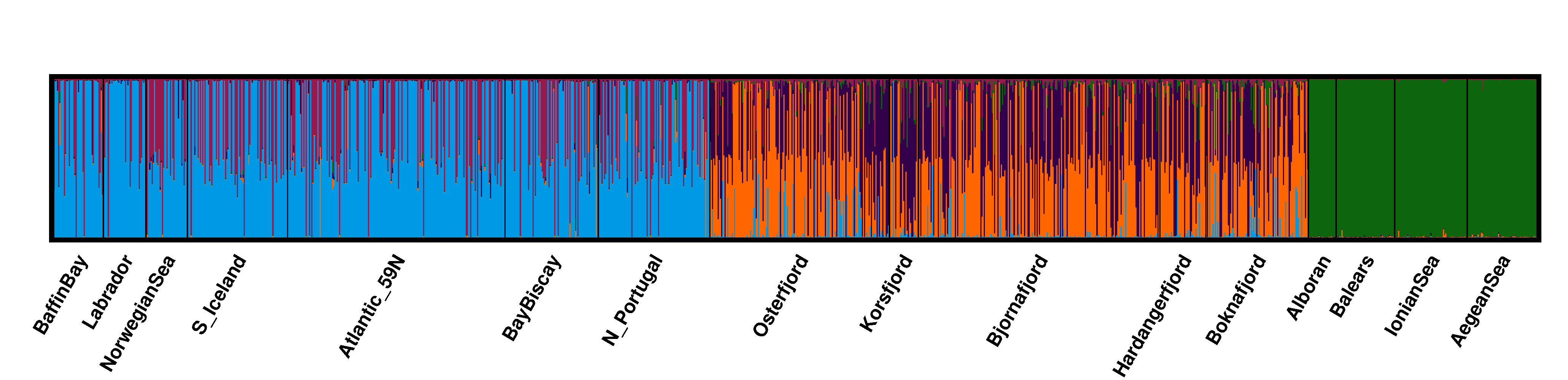 |

**Fig. S6.** Barplot representing the proportion of individuals’ ancestry to cluster at a) K3, b) K4 and c) K5 as inferred from Bayesian clustering in STRUCTURE assessed from the set of 121 SNP loci.


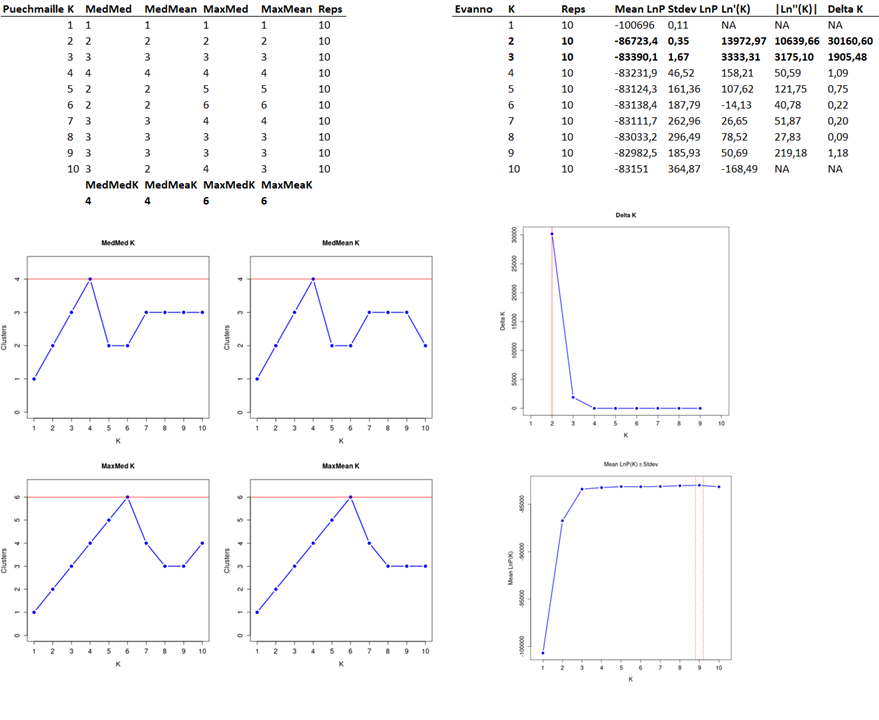


**Fig. S7.** *A posteriori* analysis of STRUCTURE outcome for the set of 84 LD-pruned loci following Puechmaille and Evanno’s statistics.

| a)  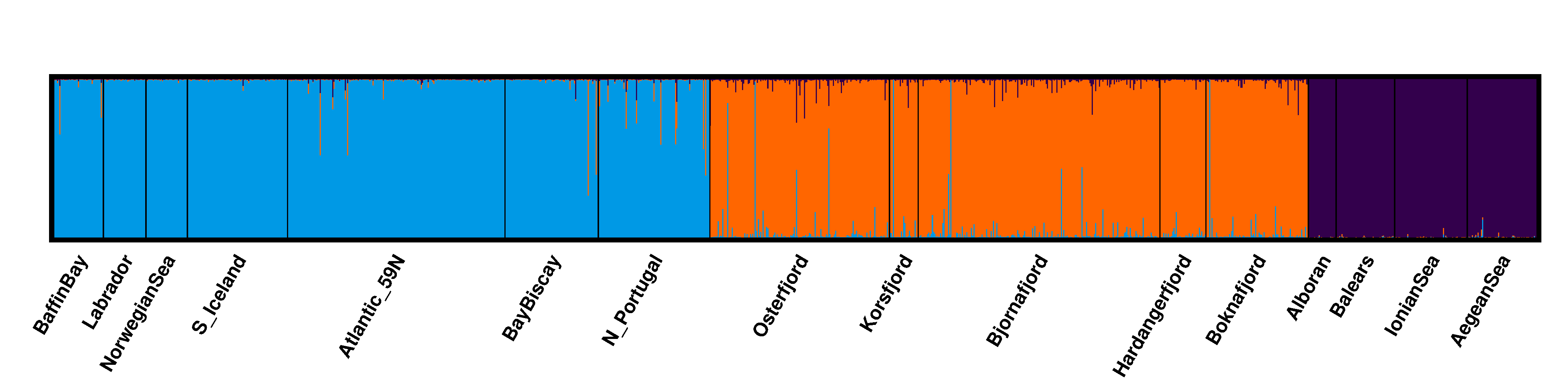 |
| --- |
| b)  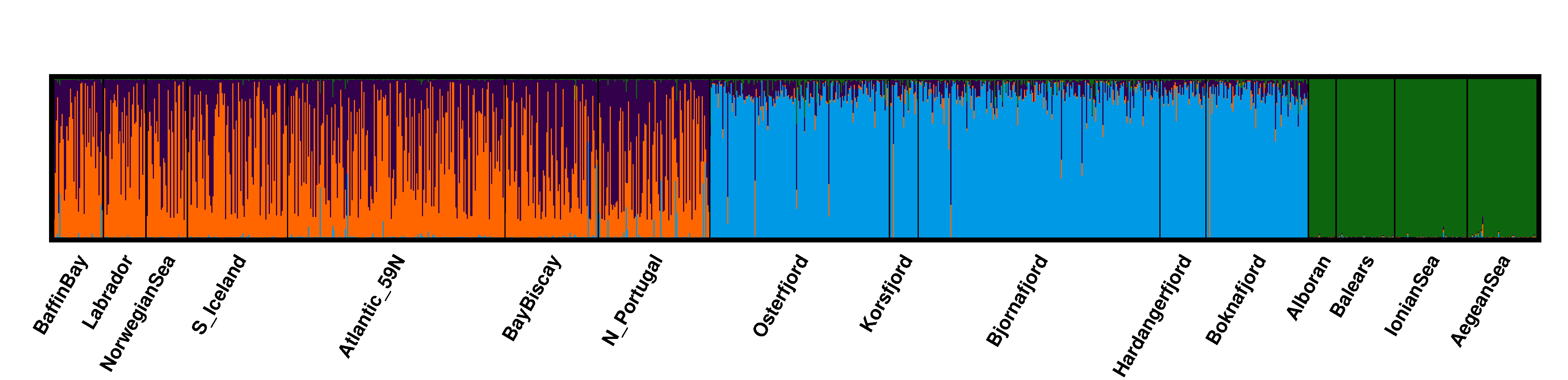 |
| c)  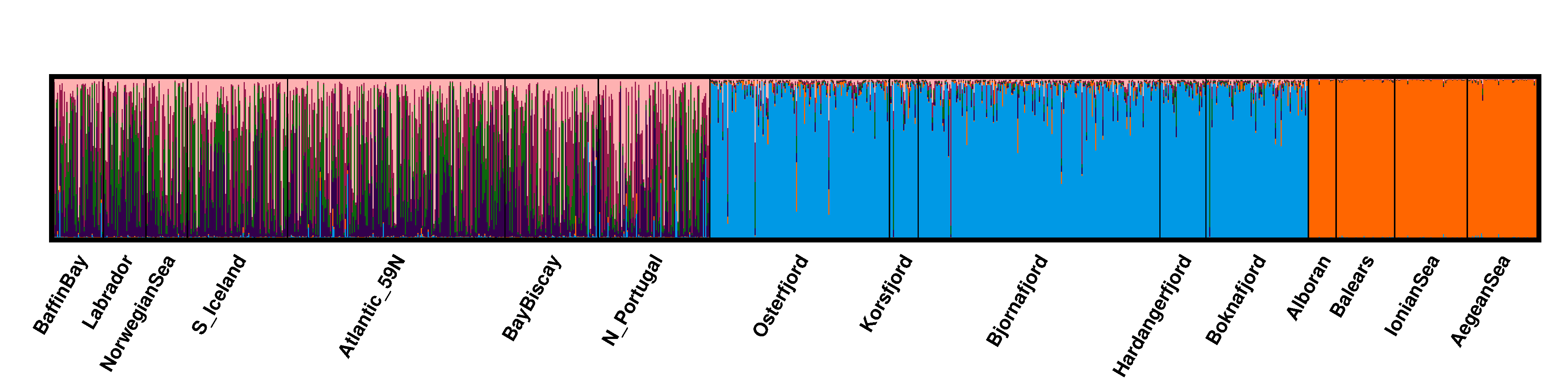 |

**Fig. S8.** Barplot representing the proportion of individuals’ ancestry to cluster at a) K3, b) K4, and c) K6 as inferred from Bayesian clustering in STRUCTURE assessed from the set of 84 LD pruned SNP loci.

| a)  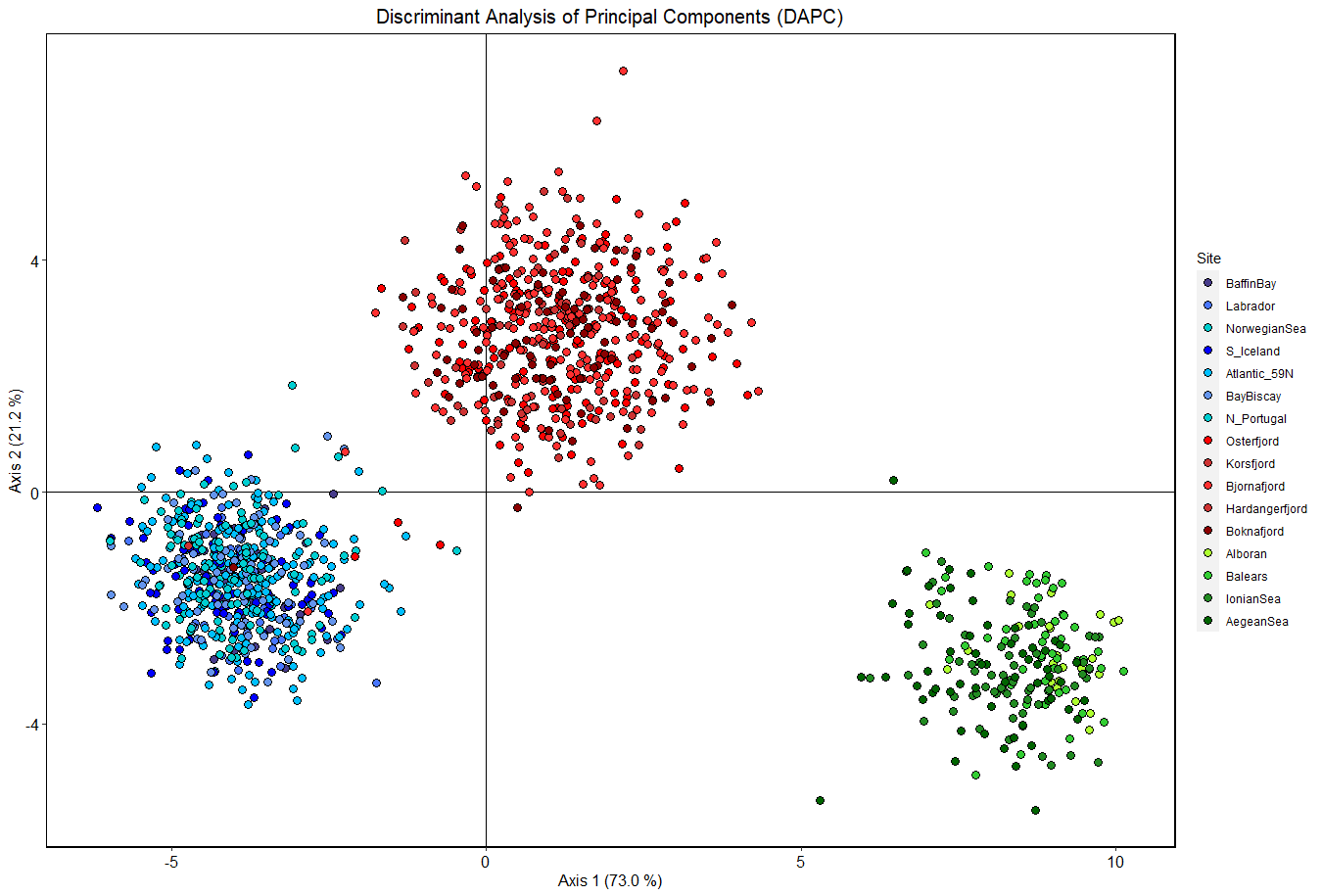 |
| --- |
| b)  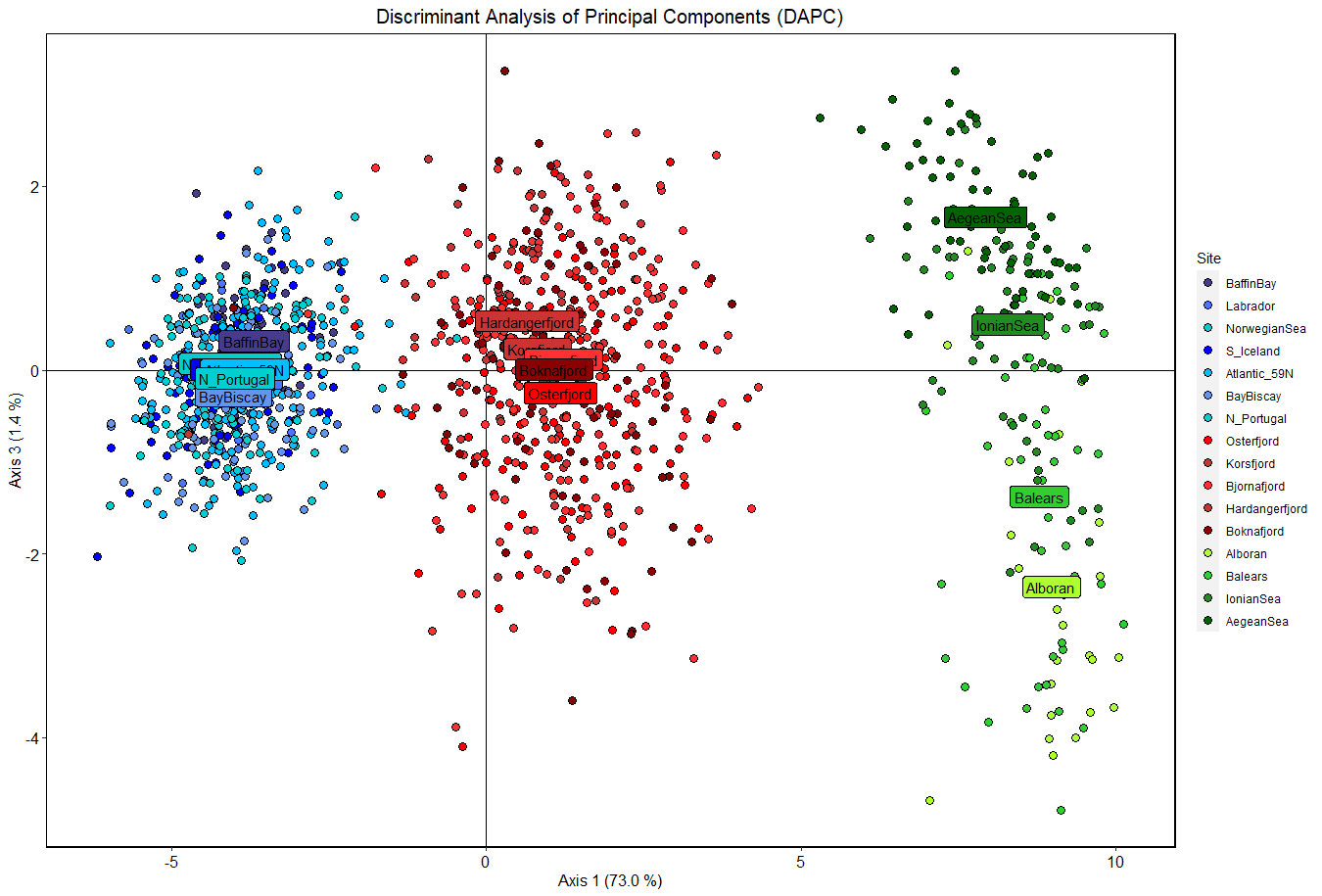 |

**Fig. S9.** Genetic differentiation among *Benthosema glaciale* samples assessed with 84 LD pruned SNP loci using Discriminant Analysis of Principal Components (DAPC) after retaining 70 principal components and 15 discriminant functions. Individuals from different sampling sites are represented by coloured dots, and name labels are centred in the mean on the inertia ellipse each geographically-explicit sample.

**Table S1.** Genetic differentiation between geographically-explicit samples calculated with the haplotypes obtained from the five most influential loci: Heatmap of pairwise *F*_ST_ values in the bottom diagonal and corresponding *P*-values after 10000 permutations in the top diagonal, with the ones significantly different from zero after FDR correction highlighted in boldface type. Greener colours indicate low differentiation increasing towards red to indicate larger differentiation.

|  |  | **OCEANIC** | | | | | | | **NORWEGIAN FJORDS** | | | | | **MEDITERRANEAN** | | | |
| --- | --- | --- | --- | --- | --- | --- | --- | --- | --- | --- | --- | --- | --- | --- | --- | --- | --- |
|  |  | **BaffinBay** | **Labrador** | **NorwegianSea** | **S_Iceland** | **Atlantic_59N** | **BayBiscay** | **N_Portugal** | **Osterfj.** | **Korsfj.** | **Bjørnafj.** | **Hardangerfj.** | **Boknafj.** | **Alborán** | **Balears** | **IonianSea** | **AegeanSea** |
| **OCEANIC** | **BaffinBay** | * | 0.200 | 0.169 | 0.737 | 0.591 | 0.457 | 0.327 | **0.000** | **0.003** | **0.000** | **0.000** | **0.000** | **0.000** | **0.000** | **0.000** | **0.000** |
|  | **Labrador** | 0.010 | * | **0.008** | 0.069 | 0.076 | 0.065 | 0.014 | **0.000** | **0.000** | **0.000** | **0.000** | **0.000** | **0.000** | **0.000** | **0.000** | **0.000** |
|  | **NorwegianSea** | 0.008 | 0.064 | * | 0.191 | 0.122 | 0.271 | 0.576 | **0.000** | 0.048 | **0.000** | **0.000** | **0.000** | **0.000** | **0.000** | **0.000** | **0.000** |
|  | **S_Iceland** | 0.000 | 0.017 | 0.005 | * | 0.875 | 0.686 | 0.444 | **0.000** | **0.000** | **0.000** | **0.000** | **0.000** | **0.000** | **0.000** | **0.000** | **0.000** |
|  | **Atlantic_59N** | 0.000 | 0.015 | 0.010 | 0.000 | * | 0.744 | 0.262 | **0.000** | **0.000** | **0.000** | **0.000** | **0.000** | **0.000** | **0.000** | **0.000** | **0.000** |
|  | **BayBiscay** | 0.000 | 0.022 | 0.003 | 0.000 | 0.000 | * | 0.544 | **0.000** | **0.001** | **0.000** | **0.000** | **0.000** | **0.000** | **0.000** | **0.000** | **0.000** |
|  | **N_Portugal** | 0.000 | 0.035 | 0.000 | 0.000 | 0.001 | 0.000 | * | **0.000** | **0.002** | **0.000** | **0.000** | **0.000** | **0.000** | **0.000** | **0.000** | **0.000** |
| **FJORDS** | **Osterfjord** | 0.180 | 0.262 | 0.097 | 0.175 | 0.192 | 0.167 | 0.139 | * | 0.294 | 1.000 | 0.321 | 0.534 | **0.000** | **0.000** | **0.000** | **0.000** |
|  | **Korsfjord** | 0.121 | 0.224 | 0.038 | 0.114 | 0.126 | 0.105 | 0.076 | 0.001 | * | 0.336 | 0.139 | 0.173 | **0.000** | **0.000** | **0.000** | **0.000** |
|  | **Bjørnafjord** | 0.177 | 0.257 | 0.096 | 0.172 | 0.188 | 0.165 | 0.137 | 0.000 | 0.001 | * | 0.301 | 0.493 | **0.000** | **0.000** | **0.000** | **0.000** |
|  | **Hardangerfjord** | 0.261 | 0.365 | 0.153 | 0.253 | 0.268 | 0.237 | 0.204 | 0.000 | 0.023 | 0.001 | * | 0.622 | **0.000** | **0.000** | **0.000** | **0.000** |
|  | **Boknafjord** | 0.214 | 0.303 | 0.121 | 0.209 | 0.227 | 0.198 | 0.168 | 0.000 | 0.010 | 0.000 | 0.000 | * | **0.000** | **0.000** | **0.000** | **0.000** |
| **MEDIT.** | **Alborán** | 0.730 | 0.824 | 0.636 | 0.666 | 0.642 | 0.646 | 0.612 | 0.319 | 0.525 | 0.313 | 0.337 | 0.313 | * | 1.000 | 1.000 | 1.000 |
|  | **Balears** | 0.792 | 0.872 | 0.719 | 0.714 | 0.670 | 0.697 | 0.660 | 0.353 | 0.636 | 0.340 | 0.425 | 0.365 | 0.000 | * | 1.000 | 1.000 |
|  | **IonianSea** | 0.812 | 0.886 | 0.745 | 0.731 | 0.680 | 0.716 | 0.677 | 0.367 | 0.670 | 0.351 | 0.456 | 0.385 | 0.000 | 0.000 | * | 1.000 |
|  | **AegeanSea** | 0.807 | 0.882 | 0.739 | 0.727 | 0.678 | 0.711 | 0.673 | 0.363 | 0.662 | 0.348 | 0.449 | 0.380 | 0.000 | 0.000 | 0.000 | * |

**Table S2.** Sample summary statistics obtained for the set of 84 LD-pruned SNP loci: Sampling sites with geographic coordinates in decimal degrees; number of individuals (N), polymorphic loci (%), observed heterozygosity, *H*_o_ (mean ± SE); unbiased expected heterozygosity, u*H*_e_ (mean ± SE); inbreeding coefficient, *F*_IS_ (mean ± SE); number of deviations from Hardy-Weinberg equilibrium (HWE) at α=0.05; number of deviations from Linkage Disequilibrium (LD) at α=0.05 both before and (after) False Discovery Rate (FDR) correction.

| **Type** | **Sample** | **Latitude** | **Longitude** | **N** | **Polym. loci (%)** | ***H*o** | ***H*e** | ***F*_IS_** | **Dev HWE (FDR)** |
| --- | --- | --- | --- | --- | --- | --- | --- | --- | --- |
| Oceanic | BaffinBay | 61.43 | -60.65 | 43 | 91.7 | 0.238 ± 0.019 | 0.256 ± 0.019 | 0.032 ± 0.025 | 11 (4) |
|  | Labrador | 65.94 | -58.13 | 37 | 85.7 | 0.235 ± 0.020 | 0.253 ± 0.020 | 0.045 ± 0.025 | 8 (2) |
|  | NorwegianSea | 63.65 | 4.10 | 36 | 84.5 | 0.240 ± 0.020 | 0.263 ± 0.019 | 0.067 ± 0.031 | 10 (5) |
|  | S_Iceland | 62.19 | -18.42 | 87 | 86.9 | 0.257 ± 0.020 | 0.266 ± 0.020 | 0.028 ± 0.017 | 7 (3) |
|  | Atlantic_59N | 59.58 | -15.68 | 189 | 94.0 | 0.257 ± 0.018 | 0.268 ± 0.019 | 0.035 ± 0.014 | 17 (10) |
|  | BayBiscay | 46.71 | -8.69 | 81 | 92.9 | 0.259 ± 0.018 | 0.271 ± 0.018 | 0.024 ± 0.018 | 10 (5) |
|  | N_Portugal | 41.53 | -12.92 | 97 | 92.9 | 0.263 ± 0.018 | 0.279 ± 0.018 | 0.050 ± 0.019 | 16 (8) |
| Fjords | Osterfjord | 60.62 | 5.52 | 156 | 100.0 | 0.306 ± 0.018 | 0.324 ± 0.017 | 0.050 ± 0.018 | 19 (10) |
|  | Korsfjord | 60.16 | 5.09 | 25 | 95.2 | 0.319 ± 0.020 | 0.329 ± 0.018 | 0.018 ± 0.025 | 10 (0) |
|  | Bjørnafjord | 60.12 | 5.62 | 210 | 100.0 | 0.308 ± 0.018 | 0.322 ± 0.017 | 0.036 ± 0.018 | 20 (12) |
|  | Hardangerfjord | 60.03 | 5.93 | 40 | 97.6 | 0.322 ± 0.019 | 0.328 ± 0.017 | 0.009 ± 0.023 | 11 (4) |
|  | Boknafjord | 59.20 | 5.61 | 89 | 98.8 | 0.308 ± 0.018 | 0.323 ± 0.017 | 0.039 ± 0.020 | 17 (7) |
| Mediterranean | Alborán | 36.00 | -3.96 | 24 | 45.2 | 0.109 ± 0.019 | 0.119 ± 0.018 | 0.059 ± 0.032 | 6 (3) |
|  | Balears | 38.50 | 2.50 | 51 | 56.0 | 0.124 ± 0.019 | 0.125 ± 0.018 | 0.015 ± 0.024 | 4 (3) |
|  | IonianSea | 38.09 | 22.74 | 63 | 52.4 | 0.106 ± 0.017 | 0.115 ± 0.017 | 0.079 ± 0.027 | 9 (6) |
|  | AegeanSea | 37.60 | 23.26 | 60 | 56.0 | 0.124 ± 0.020 | 0.122 ± 0.019 | 0.000 ± 0.019 | 4 (2) |

**Table S3.** Genetic differentiation between geographically-explicit samples estimated for the total 84 LD-pruned SNP loci: Heatmap of pairwise *F*_ST_ values in the bottom diagonal and corresponding *P*-values after 10000 permutations in the top diagonal, with the ones significantly different from zero after FDR correction highlighted in boldface type. Greener colours indicate low differentiation increasing towards red to indicate larger differentiation.

|  |  | **OCEANIC** | | | | | | | **NORWEGIAN FJORDS** | | | | | **MEDITERRANEAN** | | | |
| --- | --- | --- | --- | --- | --- | --- | --- | --- | --- | --- | --- | --- | --- | --- | --- | --- | --- |
|  |  | **BaffinBay** | **Labrador** | **NorwegianSea** | **S_Iceland** | **Atlantic_59N** | **BayBiscay** | **N_Portugal** | **Osterfj** | **Korsfj** | **Bjørnafj** | **Hardangerfj** | **Boknafj** | **Alborán** | **Balears** | **IonianSea** | **AegeanSea** |
| **OCEANIC** | **BaffinBay** | * | 0.971 | 0.482 | 0.991 | 1.000 | 0.809 | 0.853 | **0.000** | **0.000** | **0.000** | **0.000** | **0.000** | **0.000** | **0.000** | **0.000** | **0.000** |
|  | **Labrador** | 0.000 | * | 0.495 | 1.000 | 1.000 | 0.933 | 0.772 | **0.000** | **0.000** | **0.000** | **0.000** | **0.000** | **0.000** | **0.000** | **0.000** | **0.000** |
|  | **NorwegianSea** | 0.000 | 0.000 | * | 1.000 | 1.000 | 1.000 | 1.000 | **0.000** | **0.000** | **0.000** | **0.000** | **0.000** | **0.000** | **0.000** | **0.000** | **0.000** |
|  | **S_Iceland** | 0.000 | 0.000 | 0.000 | * | 0.970 | 0.113 | 0.016 | **0.000** | **0.000** | **0.000** | **0.000** | **0.000** | **0.000** | **0.000** | **0.000** | **0.000** |
|  | **Atlantic_59N** | 0.000 | 0.000 | 0.000 | 0.000 | * | 0.472 | 0.098 | **0.000** | **0.000** | **0.000** | **0.000** | **0.000** | **0.000** | **0.000** | **0.000** | **0.000** |
|  | **BayBiscay** | 0.000 | 0.000 | 0.000 | 0.002 | 0.000 | * | 0.654 | **0.000** | **0.000** | **0.000** | **0.000** | **0.000** | **0.000** | **0.000** | **0.000** | **0.000** |
|  | **N_Portugal** | 0.000 | 0.000 | 0.000 | 0.004 | 0.002 | 0.000 | * | **0.000** | **0.000** | **0.000** | **0.000** | **0.000** | **0.000** | **0.000** | **0.000** | **0.000** |
| **FJORDS** | **Osterfjord** | 0.175 | 0.181 | 0.168 | 0.183 | 0.193 | 0.174 | 0.170 | * | 0.323 | **0.000** | **0.000** | **0.000** | **0.000** | **0.000** | **0.000** | **0.000** |
|  | **Korsfjord** | 0.181 | 0.187 | 0.175 | 0.184 | 0.188 | 0.176 | 0.165 | 0.001 | * | 0.719 | 0.322 | 0.569 | **0.000** | **0.000** | **0.000** | **0.000** |
|  | **Bjørnafjord** | 0.170 | 0.175 | 0.166 | 0.177 | 0.185 | 0.171 | 0.167 | 0.007 | 0.000 | * | 0.074 | **0.003** | **0.000** | **0.000** | **0.000** | **0.000** |
|  | **Hardangerfj** | 0.194 | 0.204 | 0.193 | 0.203 | 0.208 | 0.195 | 0.182 | 0.015 | 0.001 | 0.003 | * | 0.271 | **0.000** | **0.000** | **0.000** | **0.000** |
|  | **Boknafjord** | 0.175 | 0.182 | 0.171 | 0.182 | 0.193 | 0.175 | 0.167 | 0.007 | 0.000 | 0.004 | 0.001 | * | **0.000** | **0.000** | **0.000** | **0.000** |
| **MEDIT.** | **Alborán** | 0.538 | 0.562 | 0.554 | 0.505 | 0.488 | 0.493 | 0.477 | 0.188 | 0.253 | 0.196 | 0.253 | 0.208 | * | 0.530 | **0.000** | **0.000** |
|  | **Balears** | 0.527 | 0.547 | 0.541 | 0.501 | 0.481 | 0.491 | 0.476 | 0.184 | 0.254 | 0.191 | 0.256 | 0.205 | 0.000 | * | **0.000** | **0.000** |
|  | **IonianSea** | 0.531 | 0.555 | 0.549 | 0.501 | 0.475 | 0.490 | 0.475 | 0.183 | 0.266 | 0.192 | 0.265 | 0.213 | 0.049 | 0.028 | * | **0.003** |
|  | **AegeanSea** | 0.513 | 0.537 | 0.530 | 0.484 | 0.460 | 0.474 | 0.459 | 0.173 | 0.245 | 0.179 | 0.246 | 0.197 | 0.078 | 0.046 | 0.011 | * |
